# Deep-DPC: Deep learning-assisted label-free temporal imaging discovery of anti-fibrotic compounds by controlling cell morphology

**DOI:** 10.1016/j.jare.2025.02.028

**Published:** 2025-02-24

**Authors:** Xu-dong Xing, Xiang-yu Yan, Yan-wei Tan, Yang Liu, Yi-xin Cui, Chun-ling Feng, Yu-ru Cai, Han-lin Dai, Wen Gao, Ping Zhou, Hui-ying Wang, Ping Li, Hua Yang

**Affiliations:** State Key Laboratory of Natural Medicines, China Pharmaceutical University, No. 639 Longmian Dadao, Nanjing 211198, China

**Keywords:** *deep-DPC*, Cellular morphology, Digital phase contrast imaging, Deep learning, Anti-fibrotic Drug discovery

## Abstract

**Introduction:**

Fibrosis can damage the normal function of many organs, such as cardiac function, for which no effective clinical therapies exist. However, traditional approaches to anti-fibrosis drug discovery have primarily focused on the final biological indicators, often overlooking the dynamic morphological changes during fibrosis progression. Here, we present a novel approach, *deep-DPC*, which integrates label-free, time-series digital phase contrast (DPC) imaging with cell morphology analysis and unsupervised machine learning to dynamically control and monitor cell morphology.

**Objectives:**

This method enables discrimination between resting and activated fibrocytes and facilitates the discovery of non-invasive labeled anti-fibrotic lead compounds.

**Methods:**

The *deep-DPC* comprises two major steps: (1) preliminary analysis by Harmony 4.9 software and (2) image classification *via* a neural network. For the experiment dataset, label-free time-series imaging was acquired from each well at 10 × magnification using the high-content imaging system, equipped with a high-speed charge-coupled device (CCD) camera. Dual-channel output images were generated through the imaging system, with one channel for bright-field and the other for DPC imaging, captured at 30-minute intervals. Firstly, applying the anti-fibrotic cell model as a case, a label-free time-series DPC imaging was developed by combining cell morphological analysis and deep learning, and its stability was verified by training with 12,000 images. Furthermore, the application of *deep-DPC* in the discovery of anti-fibrotic lead compounds.

**Results:**

Using the *deep-DPC* platform, over 100,000 images generated from 1,400 compounds were processed, identifying Neo-Przewaquinone A as a potent anti-fibrosis agent. Neo-Przewaquinone A exerts its effects by inhibiting TGF-β receptor I, thereby maintaining cells in a resting state and arresting the cell cycle.

**Conclusion:**

The *deep-DPC* offers a promising strategy for fibrosis assessment by combining deep learning with dynamic cell morphology analysis based on time-series DPC images. Additionally, the platform holds potential as a novel therapeutic approach for anti-myocardial fibrosis by regulating cell morphology.

## Highlights

• A strategy, *deep-DPC* combines label-free, time-series Digital Phase Contrast imaging with cell morphology analysis and deep learning to dynamically control and monitor cell morphology.
• The *deep-DPC* utilizes deep learning to discriminate between the resting (fibroblast) and activated (myofibroblast) states by “controlling” cell morphology.
• The *deep-DPC* was applied to the screening of 1400 compounds derived from natural products.
• A novel molecule-Neo-Przewaquinone A was discovered, which exhibits anti-fibrotic activity by inhibiting TGF-*β* receptor I, maintaining cells in a resting state.

## Graphical abstract

**Figure:**
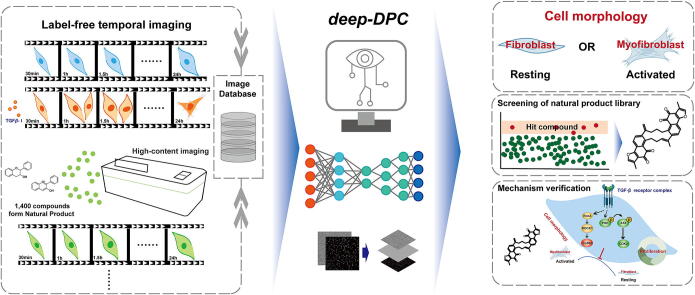

## Introduction

Fibrosis, which can occur in various diseases, leads to the excessive accumulation of fibrous connective tissue, destroying organ structures, dysfunction, and, in severe cases, organ failure. This condition poses a significant threat to human health [1], [2], [3]. Moreover, there is a notable lack of effective drugs for treating various types of fibrosis [4], [5], underscoring the urgent need for new therapeutic strategies and approaches. In particular, cardiac fibrosis is a common comorbidity in ischemic heart failure, genetic cardiomyopathies, diabetes mellitus, and aging, which exacerbates cardiac dysfunction and ultimately contributes to increased morbidity and mortality, but no effective clinical therapies exist [6]. While fibrosis was traditionally viewed as a relentlessly progressive and irreversible condition, recent preclinical models and clinical trials have revealed that fibrosis is, in fact, a highly dynamic process, involving the transition between fibroblasts and myofibroblasts. However, current anti-fibrotic drug screening methods predominantly focus on fibrotic indicators or rely on fluorescence staining, which are time-consuming and have low throughput [7]. Additionally, these methods often target the fibrotic process itself, which was mostly based on fixed-time biomarker staining methods (e.g., α-SMA or Collagen-I). However, neglecting the preservation of the original fibrocyte state, which plays a critical role in many tissues and organs [8]. Therefore, a major challenge in discovering effective therapies for fibrotic diseases is the development of robust phenotyping methods for disease model cells that are conducive to drug discovery. Compared to previous anti-fibrosis screening methods, it is necessary to accurately capture cell behavior and characteristics, provide insights into disease mechanisms, and also be suitable for high-throughput screening.

With the rapid advancements of biological imaging technology, high-content imaging (HCI) based on cellular phenotypes and morphology has become widely utilized in drug discovery [9], [10], [11], [12], chemical starting points identification, and early toxicity prediction [13], [14], [15]. However, many established studies focused on detecting cell morphology or cellular structures using multifocal fluorescence imaging, which relies heavily on biomarkers or fluorescence probes [16]. Previously, some analysis methods based on bright-field images have been established [17], [18]. However, these methods are also commonly used to detect fixed-time data and to examine the apoptotic state of cells, ignoring the dynamic changes during cell growth. In contrast, Digital phase contrast (DPC) imaging is a label-free and non-invasive technique capable of capturing dynamic cellular changes in real-time without requiring additional preparation such as immunofluorescence staining with specific antibodies [19]. Previously, some were based in part on target-free methods, such as analyses that used only white-field images to detect apoptosis.

Despite its potential, DPC image processing has generally focused on cell segmentation and detection [20], [21], also trying to use DPC images to observe cell activity trajectories [22]. However, analysis of dynamic changes in cell morphology is limited. Understanding the mechanical functions underlying cellular morphology is critical, as cell morphology and intracellular organization are shaped by the temporal and spatial regulation of gene networks within local signaling environments [23], [24], [25]. Thus, drug screening based on cell morphology is essential, particularly for diseases with complex or unclear pathogenesis. For instance, the cell painting assay uses fluorescent probes to generate hundreds of cell morphological parameters, which are then analyzed using software [26]. Given the widespread application of artificial intelligence (AI) in face and image recognition technologies [27], unbiased deep learning approaches should be increasingly adopted in chemical biology and drug discovery [28], [29]. When combined with cell morphology analysis, deep learning can significantly enhance the utility of DPC imaging in disease treatment and anti-fibrosis drug discovery.

In this study, we developed a cell morphology parameter analysis and unbiased deep learning processing method, termed *deep-DPC*, which utilizes multi-view projections for the supervised and unsupervised analysis of time-series DPC imaging. To demonstrate its utility, we applied *deep-DPC* to an anti-fibrotic cell model, combining label-free, time-series DPC imaging with cell morphology analysis and deep learning. This system was validated using 12,000 images, confirming its stability. Natural products (NPs) represent a rich resource of structurally diverse and highly effective compounds for drug discovery [30], [31]. Therefore, we aimed to identify anti-fibrotic lead compounds within a library of 1,400 NPs. Using *deep-DPC*, we processed over 100,000 images and identified Neo-Przewaquinone A (NPA) as a promising candidate for anti-fibrotic therapy. NPA exerts its effects by inhibiting Transforming growth factor beta (TGF-β) receptor I, preventing excessive fibrocyte proliferation, and regulating cell morphology. In summary, we present an artificial intelligence tool that integrates cell morphology with image-deep neural networks for DPC image-based fibrosis assessment, offering a novel strategy for anti-fibrotic therapy by targeting TGF-β signaling to control cell morphology.

## Materials and methods

### Cell lines and primary neonatal rat cardiac fibroblast (NRCFs) isolation and culture

NIH-3T3 mouse embryonic fibroblast (NIH-3T3) cells and Embryonic rat cardiomyocyte-derived (H9c2) cells were purchased from the National Collection of Authenticated Cell Cultures, Chinese Academy of Sciences (NCAC, Shanghai, China). NIH-3T3 cells were cultured in Dulbecco's Modified Eagle's Medium (DMEM, KeyGenbio, China) supplemented with 10 % of Newborn calf serum (NBCS, Thermo, USA) and 1 % of penicillin and streptomycin. H9c2 cells were cultured in DMEM (KeyGenbio, China) supplemented with 10 % of Fetal Bovine Serum (FBS, Thermo, USA) and 1 % of penicillin and streptomycin.

NRCFs were isolated from the heart tissue of 1–2 day-old Sprague Dawley (SD) rats. Briefly, cardiac tissue was cut into small pieces, washed with precooled PBS, digested in 0.1 % collagenase II (Biosharp, China) solution at 37 °C for 7–8 cycles. Cells were pre-incubated for 2 h to allow NRCFs to attached to the culture plate, after which cardiomyocytes were removed. NRCFs were then cultured in DMEM supplemented with 10 % FBS. All cells were incubated at 5 % CO_2_ atmosphere at 37 °C.

### High-content digital phase contrast imaging and data processing

**Fig. 1:**
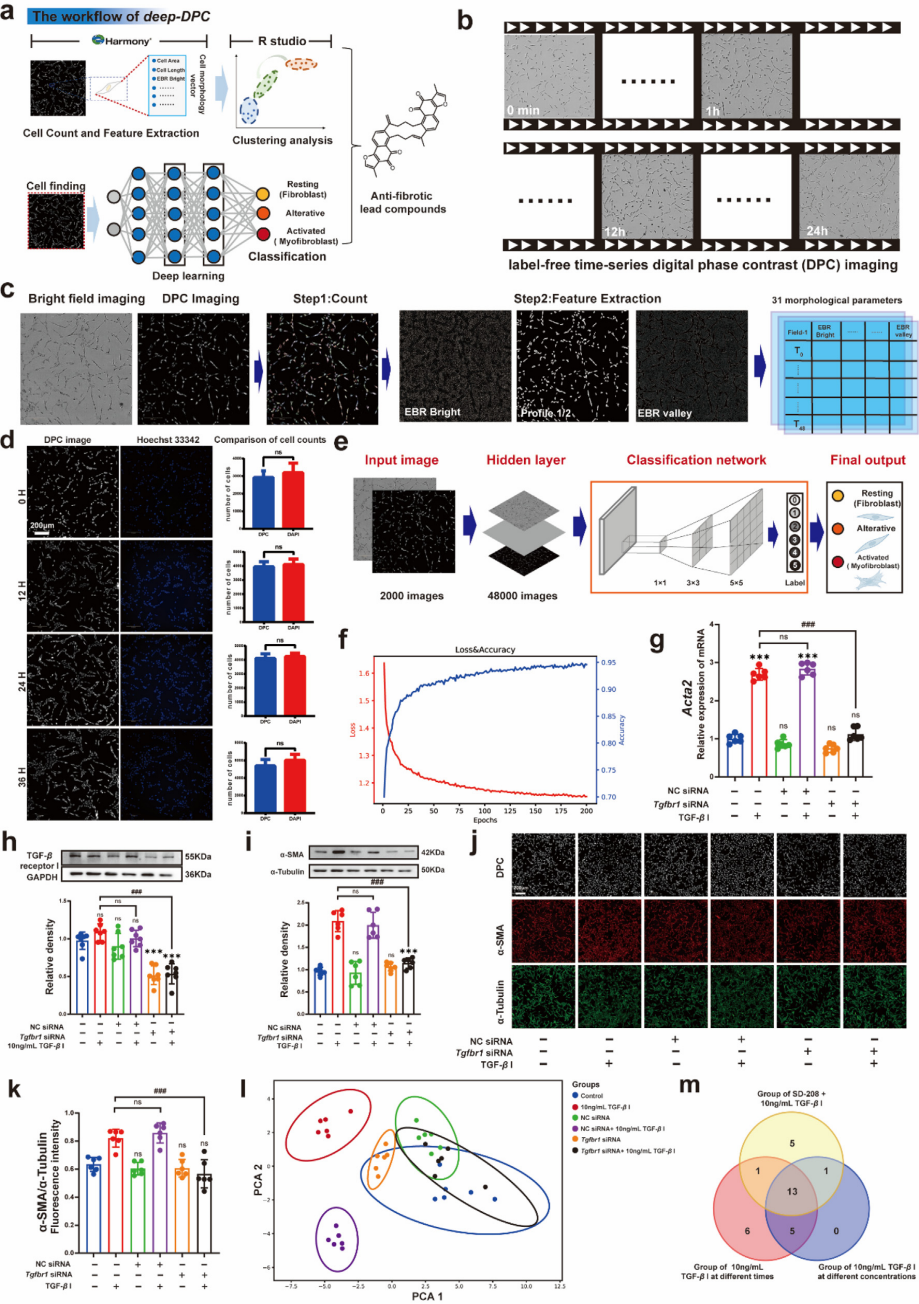
**a.** The workflow of *deep-DPC*. The combined scalable automation and deep learning to develop a label-free time-series DPC platform for unbiased scale morphological analysis of fibrosis phenotypes and drug screening. **b.** The time-series DPC imaging is done at different times every half hour by a high-content imaging system. **c.** Harmony 4.9 software analysis. **d.** DPC imaging count and Hoechst 33342 staining count were compared at 0, 12, 24, and 36 h, respectively. Scale bar: 200μ m. **e.** Prediction of anti-fibrosis by time-series DPC images based on unbiased automated deep machine learning. **f.** Tested the deep learning model on a test set with 4200 labeled images independent of the different operator's training and validation sets. **g.** The mRNA level of *Acta2* was determined by quantitative PCR (n = 6). **h.** The immunoblot analysis of TGF-β receptor I in NIH-3T3 cell (n = 6). **i.** The immunoblot analysis of α-SMA in NIH-3T3 cell (n = 6). **j.** The DPC image, the immunofluorescence of α-SMA and α-Tubulin, Scale bar: 200 μm. **k.** The fluorescence intensity ratio of the α-SMA and α-Tubulin (n = 6). **l.** Principal components in different groups analyzed the DPC images. **m.** The relationship between different groups of cell morphological feature parameters was analyzed. The results are expressed as the mean ± SD; *P < 0.05, **P < 0.01, ***P < 0.001, ^#^*P* < 0.05, ^##^*P* < 0.01, ^###^*P* < 0.001. ns, statistically not significant.

DPC images were acquired at 10 × magnification using the Opera Phenix High-Content Imaging System (Revvity). Each image had a resolution of 1360 × 1024 pixels, with 25 fields covering one well. The imaging system generated three output channels: bright-field, DPC and fluorescence imaging. The proposed deep learning and unbiased screening method integrate commercial software processing parameters with a neural network-based image analysis pipeline, including “machine learning” segmentation, preprocessing modules, and classification networks to discriminate compound activity (Fig. 1a). Data analysis was performed using Harmony 4.9 software. The software quantified the cell morphology parameters, which were extracted using a built-in algorithm. A total of 31 morphological parameters of NIH-3T3 cells were obtained through the analysis of DPC images using Harmony 4.9 software. These morphological parameters and α-smooth muscle actin (α-SMA) fluorescence intensity were analyzed for subsequent experiments. Linear regression analysis was employed to assess the correlation between DPC image parameters and α-SMA protein fluorescence intensity. The linear correlation analysis was performed between DPC parameters and α-SMA protein expression to identify relationships.

### The coefficient of variation (CV) and Z' factor

Robustness and reproducibility of high throughput screening on the 96-pillar plate were established by the Z′ factor [32] and CV.

The CV is the ratio of the standard deviation to the average (Ave).

$$\mathrm{CV}=\frac{\mathrm{SD}}{\mathrm{Ave}}\times 100\%$$

Meanwhile, the Z’ factor was calculated using the following equation:

$$Z^{\prime }-factor=1-\frac{3\times (\sigma_{m}+\sigma_{c})}{|\mu_{m}-\mu_{c}|}$$

where $\mu_{m}$ is the average growth ratio of each plate in the model group (10 ng/mL TGF-*β* I), $\mu_{c}$ is the average growth ratio of each plate in the control group. $\sigma_{m}$ is the standard deviation of each plate in the model group (10 ng/mL TGF-β I), and $\mu_{c}$ is the standard deviation of each plate in the control group.

### The architecture of deep-DPC

Fig. 1a illustrates the workflow of *deep-DPC*. The process consists of two stages: Process 1 involves data processing using Harmony 4.9 software and R studio. Process 2 applies the Inception V4 architecture neural network to classify the images. The initial input was a 16-bit image containing bright field and DPC channels. Each channel of the original image was resized and normalized, and a third channel was generated by averaging the two existing channels to form an RGB image. This 299 × 299 × 3 image was then input into the Inception V4 network. The output dimension of the penultimate fully connected layer was modified to six catelogies, enabling classification of cell images into six types. The Inception V4 architecture was pre-trained on the ImageNet object recognition dataset, which consists of 1.2 million non-cell images across 1,000 categories. Pre-trained convolutional layers were used and concatenated with randomly initialized fully connected layers, then trained the resulting network on our custom dataset. TGF-β I was used as the modeling agent, and various concentrations of TGF-β I were added to NIH-3T3 cells. Images of cells treated with vehicle were labeled as type 0, while images of cells treated with 0.01, 0.10, 1, 5, 10 or 20 ng/mL of TGF-β I were labeled as type 1–5. We defined type 0 and 1 cell as normal (un-activated) cell, types 2 and 3 as alternative cells, and types 4 and 5 as fibrotic cells. A dataset containing 24,000 labeled images, equally distributed across the six types, was created. The dataset was randomly split into training and validation sets with a ratio of 4:1, resulting in 19,200 training images and 4,800 validating images. Softmax cross-entrophy was used as the loss function, the optimizer was Adam.

### Screening of natural product library

A high-throughput drug screening platform was established to identify compounds capable of inhibiting fibrosis, performed in a 96-well format as described in this study. Briefly, NIH-3T3 cells were seeded in Cell-Carrier 96-well plates (Revvity) at a density of 3,000 cells per well in 100 μ L of medium and incubated at 37° C. Cell counts were performed as described above prior to drug administration. Then, 10 μL of natural product (NP) compounds, dissolved in DMSO and pre-diluted in DMEM were added to achieve a final concentration of 10 μM in each well.

### Interactions between small molecules and TGF-β receptor 1

Surface plasmon resonance (SPR) experiments were conducted using a Biacore T200 (Cytiva, USA). The CM5 sensor chip surface was prepared, and TGF-β receptor I was immobilized on the chip *via* an EDC/NHS cross-linking reaction. TGF-β receptor I was optimized in different pH sodium acetate solutions, with a final concentration of 30 μg/mL in 10 mM acetate (pH 4.5). NPA was diluted in 0.01 % DMSO in PBS-P buffer at a series of concentrations and injected over the immobilized TGF-β receptor I at a flow of 30 μ L/min. The contact time was set to 60 s, followed by a dissociation time of 150 s. Reference channels without immobilized protein were used as controls. Data were analyzed with Biacore T200 Evaluation Software to calculate *K_D_* value.

### Western blotting

All cells were lysed in Red Loading Buffers (Cell Signaling Technology, USA), and total proteins were quantified using the BCA Protein Assay Kit (Beyotime, China). Equal amounts of protein were separated by SDS-PAGE and transferred to nitrocellulose (NC) membranes. The membranes were immunoblotted with primary antibodies e.g., anti-α-SMA (ab32575), anti-α-Tubulin (66031), anti-GAPDH (ab181602), anti-Collagen I (ab34710), anti-Collagen III (ab7778), anti-Phospho-PI3 Kinase p85 (Tyr458) (4228P), anti-PI3 Kinase p85 (4257P), anti-Phospho-Akt (Ser473) (9271S), anti-Akt (9272S), anti-ROCK1 (21850–1-AP), anti-RHOA Polyclonal (10749–1-AP), anti-CDK2 Antibody(60312–1-Ig), anti-Cofilin Polyclonal (10960–1-AP) overnight at 4 °C, and the membrane were incubated with HRP-labeled secondary antibodies, and protein bands were visualized using a Pierce™ Fast Western Kit (Tanon, China). ImageJ software was used to quantify band intensities.

### Immunofluorescence staining

NIH-3T3 cells or NRCFs were fixed with 4 % paraformaldehyde (Biosharp, China) in PBS for 15 min, followed by permeabilization with 0.1 % Triton X-100 (Beyotime, China) in PBS for 15 min. Samples were blocked with 10 % bovine serum albumin (BSA, Sigma-Aldrich) for 30 min, then incubated with primary antibodies overnight at 4 °C. After washing, FITC-conjugated secondary antibodies were applied for 2 h at room temperature. DAPI (Thermo, USA) was used to stain cell nuclei, and images were captured using High Content Imaging (HCI) at 10 × magnification.

### Cytotoxicity assay and lactate dehydrogenase

Cytotoxicity was measued using a CCK-8 assay kit (DOJINDO, China) following the manufacture’s protocol. Absorbance was measured with a microplate reader (BMG POLARstar Omega). The % cytotoxicity was calculated by subtracting the spontaneous release control (PBS-treated) from the lysis buffer-treated samples, normalized to NIH-3T3 cells. Technical replicates were averaged.

### Animal experiments

All animal experiments followed ethical guidelines and approved by China Pharmaceutical University (022–12-055). C57BL/6N wild-type male mice (8–10 weeks old, Vital River Laboratory Animal Technology Co., Ltd., China) were subcutaneously implanted with osmotic micropumps (ALZET®) to deliver Angiotensin II (Ang II) at 3 mg/kg/ day. Mice were fed a standard diet, maintained on a 12-hour light/dark cycle at 25 °C with libitum food and water. General anesthesia was induced with isoflurane (5 % isoflurane and 95 % oxygen, flow of 5 L/min). In the therapeutic study, Ang II was administered *via* osmotic pumps (ALZET micro-osmotic pump model) for 8 weeks (re-implantation in 4 weeks). NPA was injected at doses of 5, 10, and 20 mg/kg.

Cardiac function was evaluated 8 weeks post-surgery using echocardiography (Vevo 3100LT, Fujifilm/VisualSonics, Canada). The parasternal short-axis and long-axis views of the left ventricle were recorded in B-mode and M−mode, respectively. Doppler imaging was performed separately using the apical four-chamber view, and all echocardiographic data were analyzed using Vevo LAB software. The MPI was calculated as the sum of isovolumic contraction time (ICT) and isovolumic relaxation time (IRT), divided by ejection time (ET).

At the end of the experiment, animals were sacrificed, and hearts were collected for histology, UPLC-QTRAP-MS, and biochemical analysis. Plasma, liver, and kidney samples were collected for toxicological evaluation.

### NPA quantification by UPLC-QTRAP-MS

Quantification of NPA was using an AB SCIEX QTRAP 5500 Ultra-performance liquid chromatography-tandem time-of-flight mass spectrometer (UPLC-QTRAP-MS). Chromatography was carried out on an Agilent ZORBAX Eclipse Plus C18 column (2.1 × 150 mm 1.8 μm) with water (A) and acetonitrile (B), both contained 0.1 % formic acid. Samples (50 μL plasma, >25 mg tissue) were prepared with acetonitrile/methanol (1:1, v/v), centrifuged (10 min at 4 °C, 12000 rpm) and reconstituted in 100 μL of 50 % water-methanol containing 10 ng/mL chloramphenicol (Internal standard). 10 μL of the reconstituted sample was injected into the UPLC-QTRAP-MS for analysis.

### Biochemical analysis of plasma

Plasma samples were extracted and entrifuged at 1000g for 20 min. The levels of creatinine (CRE, C011-2–1, Nanjing Jiancheng Bioengineering Institute), glutamate-pyruvate-transaminase (GPT, C009-2–1, Nanjing Jiancheng Bioengineering Institute) and glutamate–oxaloacetate-transaminase (GOT, C010-2–1, Nanjing Jiancheng Bioengineering Institute), and urea were measured photometrically (UREAL, ab234052). A kinetic urease test with glutamate dehydrogenase (GLDH, A125-1–1, Nanjing Jiancheng Bioengineering Institute) was performed using the BMG POLARstar Omega analyzer system.

### Transcriptome sequencing and analysis

Gene expression profiling was performed by Shenzhen BGI CO., Ltd, using the HiSeq 4000 platform (BGI-Shenzhen, China). NRCFs were treated with NPA and TGF-β I for 24 h at 37 °C, and total RNA was extracted using VeZol Reagent (Vazyme, R411-01, China). The RNA was purified using a NanoDrop spectrophotometer and Agilent 2100 bioanalyzer (Thermo, USA) to access biotin-labeled cDNA. Single-end sequencing of the amplified flow cytometer on a HiSeq4000 platform (BGI Shenzhen, China).

The sequencing data were filtered and reads containing adapters were removed using SOAPnuke (v1.52). Gene expression heatmaps were generated using pheatmap (v1.0.8). Differential expression analysis was conducted using DESeq2 (v1.4.5), with Q value ≤ 0.05. To interpret changes in phenotypes, KEGG (https://www.kegg.jp/) and GO (https://www.Geneontology.Org/) enrichment analyses were performed on the annotated differentially expressed genes using the Hypergeometric test implemented in Phyper. The Bonferroni correction was applied to adjust for multiple testing, with Q ≤ 0.05 as the threshold for statistical significance. Peotien-protein interaction (PPI) networks were conducted using STRING (search tool to retrieve interacting genes/proteins).

### Cell cycle analysis

Cells were processed for flow cytometry. Approximately 1 × 10^6^ cells were pelleted by centrifugation at 2500 rpm for 10 min. After removing the supernatant, 600 μL of cold 70 % ethanol was slowly added to the pellet with vigorous mixing. For flow cytometry analysis, the ethanol was removed by centrifugation, and cells were washed once with 500 μL PBS. Subsequently, 500 μL of PI (Keygen) was added to the cell, and samples were incubated in the dark for 30 min prior to analysis. Forward light scattering (FSC) and side light scattering (SSC) were measured using a flow cytometer (Beckman, USA) with excitation at 488 nm. DNA histograms, generated without gating, were used to determine the percentages of cells with different DNA content. The DNA content of cells in exponential growth phase was analyzed by flow cytometry, and the histograms was processed using FlowJo software to quantify the proportion of cells in each phase of the cell cycle.

### Statistics

GraphPad Prism 9.0 was used for statistical analysis, including the construction of histograms, scatter plots, bar plots, and box-and-whisker plots. A two-sided *t*-test was used to compare differences between groups.

## Results and discussion

### DPC image data Acquisition and processing

Cell morphology is a critical trait in various biological contexts, particularly in disease studies or drug discovery [12], [33]. Unlike target-based approaches, cell phenotype analysis maintains disease-associated proteins in their natural cellular environment, leading to more physiologically relevant outcomes [34], [35]. Here, we developed a novel integrated cellular morphology analysis and unbiased, automated deep learning-assisted method for discovering anti-fibrotic lead compounds, termed *deep-DPC* (Fig. 1a). The workflow is composed of two major steps: (1) preliminary analysis by Harmony 4.9 software and (2) image classification *via* a neural network. For the experiment dataset, label-free time-series imaging was acquired from each well at 10 × magnification using the high-content imaging system (Opera Phenix, Revvity), equipped with a high-speed charge-coupled device (CCD) camera. Dual-channel output images were generated through the imaging system, with one channel for bright-field and another for DPC imaging, captured at 30-minute intervals (Fig. 1b).

The NIH-3T3 cells were used as a model for antifibrotic screening. Moreover, TGF-β I is closely associated with tissue fibrosis [36]. To optimize the concentration of TGF-β I for the experiment, NIH-3T3 cells as a model for antifibrotic screening were imaged using the DPC technique under different concentrations of TGF-β I (200, 100, 50, 25, 10, 5, 2.5 and 1.25 ng/mL), with images captured every 30 min (Fig. S1 a and b, Supporting Information). Then, the above image datasets are processed through the following workflow of *deep-DPC*, which can be briefly described as:

**(1) Preliminary Image Analysis.** Harmony 4.9 software was used to segment the DPC images and extract 31 morphological parameters, including features like EBR Bright and Profile 1/2 etc. (Fig. 1c). From this, a 31 × 48 temporality matrix was obtained for each field. The relative proliferation curve was generated to quantify NIH-3T3 cell proliferation over 36 h (Fig. S1a and b, Supporting Information). The results showed significant cell proliferation at 24 h under the stimulation of 10 ng/mL TGF-*β* I compared to the control group (Fig. S1b and c, Supporting Information). Additionally, the data revealed that higher concentrations of TGF-β I caused weaker proliferation (Fig. S1b, Supporting Information), suggesting bidirectional regulation effects depending on the stimulus [37], [38]. To further validate the DPC imaging parameters, we compared cell counts obtained from DPC imaging with those from fluorescence-labeled nuclei using Hoechst 33342. Three-channel output images (DPC, bright-field, and fluorescence) were generated, and cell counts were compared at 0, 12, 24, and 36 h (Fig. 1d). No significant difference was observed between the DPC and Hoechst 33342 counting methods, indicating that temporal-DPC imaging can reliably quantify cell proliferation without the need for labeling (Fig. 1d). **(2) Image Classification with Deep Learning.** The second step involved using a deep learning-based image classification method to screen the lead compounds (Fig. 1e). DPC images of cells treated with different concentrations of TGF-β I was labeled into six categories (type 0–5). Type-0 images, derived from the control group, were labeled as normal fibroblasts by the neural network. Images from the low-concentration TGF-β I group (1.25 and 2.5 ng/mL) at 12 h were classified as type-1, 2, or 3. Images from the higher-concentration of TGF-β I group (25 ng/mL and 50 ng/mL) taken after 20 h were classified as type-4 and type-5, representing activated myofibroblasts (Fig. 1e). A total of 2,000 images were labeled for each type, and data augmentation (image rotation) was applied to create a final dataset of 48,000 images. The Inception V4-based classification network was fine-tuned using this dataset. After 120 training epochs, the network’s loss function converged, and accuracy reached 95 % in both training and validation sets (Fig. 1f). The model was then tested on 4,200 independent images, achieving a prediction accuracy of approximately 92.3 %, demonstrating excellent performance on non-training data (Fig. 1f). Moreover, to prove Inception V4′s superiority, we’ve actually tested several other models, including Inception-ResNet V2, ResNet-101, and VGG-19. A total of 12,000 images were used to train the candidate classifiers. The images were evenly spread in every category. The whole dataset was divided into 5 folds, each had 2,400 images. We ran a 5-fold cross-validation on every classification model and calculated the mean precision, recall, and F1 score and their standard deviation. Under similar training conditions, the deep DPC is part of the Inception V4, which achieved the highest accuracy on our dataset, so we selected Inception V4 as our final classification model (Table S1, Supporting Information).

### Relationship between TGF-β I and dynamic changes in cell morphology analyzed through DPC imaging

Single-cell morphology is regulated by spatiotemporal signaling protein activity, which coordinates the dynamic processes involved in cellular shape and function [39], [40]. Here, we investigated the role of TGF-β I in regulating cell morphology in a fibrosis model. To explore the relationship between TGF-β I and cell morphology, DPC imaging analysis was conducted following gene interference with TGF-β receptor I (*Tgfbr1*). First, to confirm the role of TGF-β I in inducing α-SMA activation, small interfering RNA (siRNA) was applied targeting TGF-β receptor I. The siRNA targeting *Tgfbr1* (from Shanghai GenePharma Co., Ltd) was selected based on its knockdown efficiency (siRNA sequence was listed in Table S2, Supporting Information), validated by quantitative real-time PCR (Fig. S1d and e, Supporting Information). The siRNA with the highest efficiency and specificity was used for further experiments. In the *Tgfbr1* knockdown group, stimulation with 10 ng/mL TGF-β I did not lead to upregulation. As shown in Fig. 1g, *Acta2* (α-SMA) expression was significantly downregulated following *Tgfbr1* knockdown, compared with the 10 ng/mL TGF-β I-treated group. Additionally, TGF-β receptor I (TGFBR I) protein levels were also reduced after *Tgfbr1* knockdown (Fig. 1h), and no upregulation occurred in response to 10 ng/mL TGF-β I. Similarly, α-SMA protein expression was significantly decreased in the *Tgfbr1* knockdown group (Fig. 1i). In summary, knockdown of TGF-β receptor I prevented fibrosis-associated morphological changes, as demonstrated by DPC imaging analysis (Fig. 1k, j and i). Previous studies have linked TGF-β signaling pathways to cell morphology changes during disease progression (Fig. S2 and S3, Supporting Information), yet the role of these changes have not emphasized [5], [41].

To analyze the correlation between α-SMA expression and morphological parameters derived from DPC imaging, both DPC and immunofluorescence imaging were performed on the *Tgfbr1* knockdown and control groups (Fig. 1j). Immunofluorescence results were consistent with the DPC imaging analysis, showing that *Tgfbr1* knockdown affected α-SMA expression without altering α-Tubulin levels (Fig. 1j and k). Furthermore, the levels of RhoA/ROCK1/Cofilin proteins, which regulate cytoskeleton formation and cellular morphology, were unchanged between the *Tgfbr1* knockdown and 10 ng/mL TGF-β I stimulated groups (Fig. S1f–i, Supporting Information).

Next, we assessed whether *Tgfbr1* knockdown affected the morphological changes observed in DPC imaging. Principal component analysis (PCA) revealed significant changes in the morphological parameters of DPC images in the TGF-β I stimulated group (Fig. 1l). However, following *Tgfbr1* knockdown, the morphological parameters of the TGF-β I stimulated group showed no significant difference compared to the control group (Fig. 1l and Fig. S2a, Supporting Information). Since α-SMA plays a central role in fibrosis by promoting cell proliferation [1], we analyzed the relationship between DPC imaging and α-SMA protein expression by screening morphology parameters. Different concentrations of TGF-β I were used, and both DPC imaging parameters and α-SMA fluorescence intensity (Fig. S3a, Supporting Information) followed the same trend for parameters such as cell area, roundness, width, length, and aspect ratio (Fig. S3b and c, Supporting Information). Additionally, α-SMA fluorescence intensity increased with higher concentrations of TGF-β I (Fig. S3d, Supporting Information). By analyzing 31 morphologic parameters of NIH-3T3 cells obtained from DPC imaging, we found that parameters such as Cell Area, Cell Eadial Mean, and 24 others (r^2^ > 0.3) were correlated with α-SMA expression (Fig. S4, Supporting Information). To further validate these findings, we performed DPC and α-SMA fluorescence imaging at different time points using 10 ng/mL TGF-β I (Fig. S5a, Supporting Information). The DPC imaging parameters showed consistent trends with α-SMA fluorescence for the same morphological features, confirming the correlation between cell morphology and α-SMA expression over time (Fig. S5b and c. Supporting Information). Subsequent experiments focused on analyzing these morphological parameters in relation to α-SMA fluorescence intensity (Fig. S6, Supporting Information). The DPC imaging parameters including Cell Area, DPC SER Bright 0 px, along with 20 others, were significantly correlated with α-SMA protein expression (r^2^ > 0.3). A selective TGF-β receptor I inhibitor, SD-208, was then tested at concentrations of 50, 25, 10, 5, and 1 nM on NIH-3T3 cells to explore relationship between cell morphology and TGF-β signaling pathway [42], [43]. Immunofluorescence analysis showed a concentration-dependent decrease in α-SMA expression (Fig. S7a–b, Supporting Information). DPC imaging analysis revealed that 16 morphological parameters, including Cell Area and DPC SER Rright 0 px, were correlated with α-SMA fluorescence intensity under different concentrations of SD-208 treatment (r^2^ > 0.3, Fig. S8, Supporting Information).

A linear correlation analysis was performed between DPC parameters across varying TGF-β I concentrations (Fig. S4, Supporting Information), at different time points (Fig. S6, Supporting Information), and under SD-208 inhibition (Fig. S8, Supporting Information). Thirteen common DPC parameters were identified across these conditions (Fig. 1m, Table S3, Supporting Information), which will be used for further analysis of disturbance mechanism. Our linear correlation analysis between DPC parameters under various concentrations of TGF-β I (Fig. S4, Supporting Information), at different times (Fig. S6, Supporting Information), and with the TGF-β receptors I inhibitor SD-208 (Fig. S8, Supporting Information), identified 13 common disturbance parameters (Fig. 1m, Table S3, Supporting Information). This highlights the utility of phenotypic screening, which preserves protein context, yielding more translatable physiological outcomes [34], [35].

The findings discussed above highlight the intricate relationship between TGF-β I signaling and dynamic changes in cellular morphology, as revealed through DPC imaging and the regulation of α-SMA expression. This correlation between cell morphology and fibrosis markers emphasizes the potential of DPC imaging in identifying phenotypic changes during fibrosis progression. The previous methods for identifing anti-fibrosis compound typically rely on immunofluorescence or fluorescence probes, which only capture static snapshot of cellular states, our approach enables dynamic analysis. Although earlier methods suggested the potential of computational approaches for analyzing biomarker images [44], none have specifically validated their applicability to DPC imaging as in our work. Thus, we explored emerging methods for systematically and unbiasedly quantifying cell morphology to construct a dynamic landscape of cell identity.

### Application of deep-DPC in the discovery of anti-fibrotic lead compounds

**Fig. 2:**
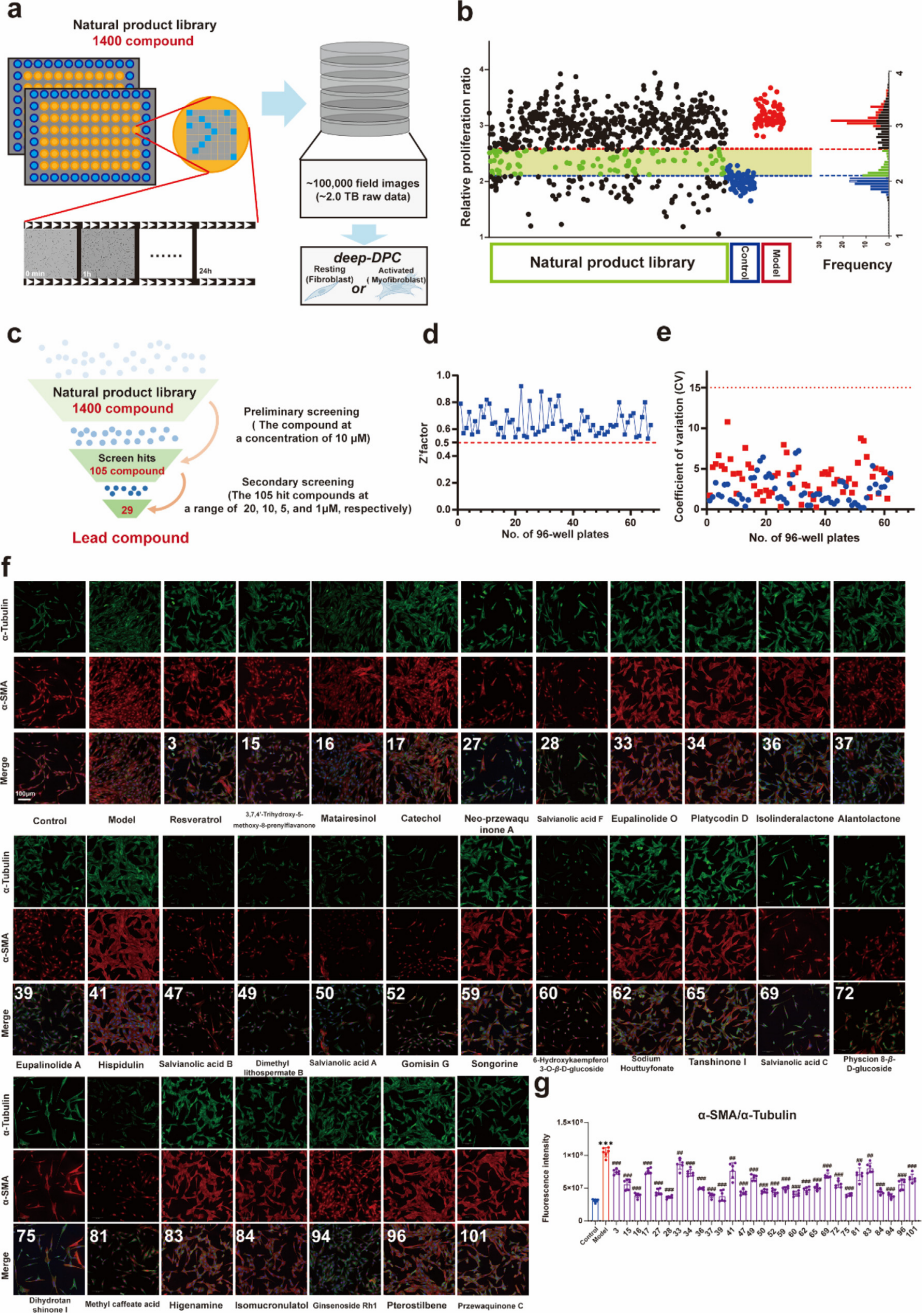
**a.** Screening of natural product library by *deep-DPC*. **b.** Screening of antifibrotic lead compounds from natural product library is a statistical method of cumulative distribution. **c.** 29 hit compounds for treating fibrosis were rapidly screened from 1400 compounds by two-step. **d.** All screening sets by Z’ factor was analyzed, indicating feasibility and reproducibility. **e.** All screening sets by coefficient of variation were analyzed, which indicates screening feasibility and reproducibility. **f.** The immunofluorescence of α-SMA (red) and α-Tubulin (Green), Scale bar: 100 μm. **g.** The fluorescence intensity ratio of the α-SMA and α-Tubulin (n = 6). The results are expressed as the mean ± SD; *P < 0.05, **P < 0.01, ***P < 0.001, ^#^*P* < 0.05, ^##^*P* < 0.01, ^###^*P* < 0.001. ns, statistically not significant.

Building upon these insights, we apply the *deep-DPC* system in a large-scale screening of natural compounds to discover potential anti-fibrotic agents. The *deep-DPC* system was tested using a self-made natural product library consisting of 1,400 compounds from traditional Chinese medicines (TCMs) to identify potential anti-fibrotic agents. The TCMs have gained increasing attention for their potential in modern drug discovery, especially in preventing and treating fibrosis [37], [38]. Our natural product library, derived from TCMs, demonstrated the potential of these compounds for anti-fibrotic drug discovery. Over 100,000 full-field images (approximately 2.0 TB of raw data, Fig. 2a) were acquired and processed through the *deep-DPC* system. All compounds were scored by *deep-DPC* system for their efficacy in improving fibrosis. The DPC images were categorized into types 0–5 at 24 h using an unbiased image learning method, identifying 105 compounds as type 0–3, which indicated their potential to delay fibrosis progression. As shown in Fig. 2b, a cumulative distribution statistical method to was employed to exclude data points representing cell proliferation in the control group (within the 95 % confidence interval) and identify compounds that effectively inhibited the cell proliferation (within 5 % confidence interval of model group). From this analysis, 105 hits were identified and further analyzed, with their names and CAS Registry Numbers listed in Table S4 (Supporting Information).

Next, 105 hit compounds were further analyzed using optimized cell morphological parameters in different concentrations (20, 10, 5, and 1 *μ*M) by the *deep-DPC* system. Simultaneously, the PCA was employed to cluster these compounds based on multidimensional parameters from *deep-DPC* system and Harmony 4.9 software by R studio, predicting that these 33 compounds might regulate α-SMA-related phenotypes and contribute to anti-fibrotic effects (Fig. S9, Supporting Information). Of these, 29 *hits* were identified using both morphological analysis and deep learning methods (Fig. 2c). The names and CAS numbers of these 29 hits are listed in Table S4 (Supporting Information). To test the stability and reliability of our screening method, we adopted two common (Z' factor and coefficient of variation, CV) used high-throughput screening system evaluation methods. The Z' factor analysis for the composite screening dataset demonstrated that the screening method was highly feasible and reproducible (Z' > 0.5, Fig. 2d). Additionally, the CV was within the expected theoretical range (Fig. 2e), further validating the reliability of *deep-DPC* method in effectively identifying potential anti-fibrotic compounds.

Interestingly, the number of compounds identified through the image-based deep learning method was higher than those identified through the morphology-based approach. This could be due to the deep learning method capturing cellular morphology changes that were not solely dependent on TGF-β I-induced mechanisms. Importantly, these 29 hit compounds were found to maintain fibrocyte morphology and prevent abnormal proliferation, aligning with the goal of screening anti-fibrosis lead compounds (As shown in Fig. 2f and g). In IC_50_ toxicity tests conducted on NIH-3T3 cells, all 29 hit compounds exhibited anti-fibrotic effects at non-toxic doses (Fig. S10, Supporting Information). Through this method, we efficiently identified 29 hits from 1,400 natural products that both maintained fibrocyte status and inhibited cell proliferation (Fig. 2e). To further validate the anti-fibrotic effects of these compounds, α-SMA protein expression was measured using immunofluorescence assay. The results showed that the 29 hit compounds significantly inhibited α-SMA expression compared with the model group (Fig. 2f and h).

**Fig. 3:**
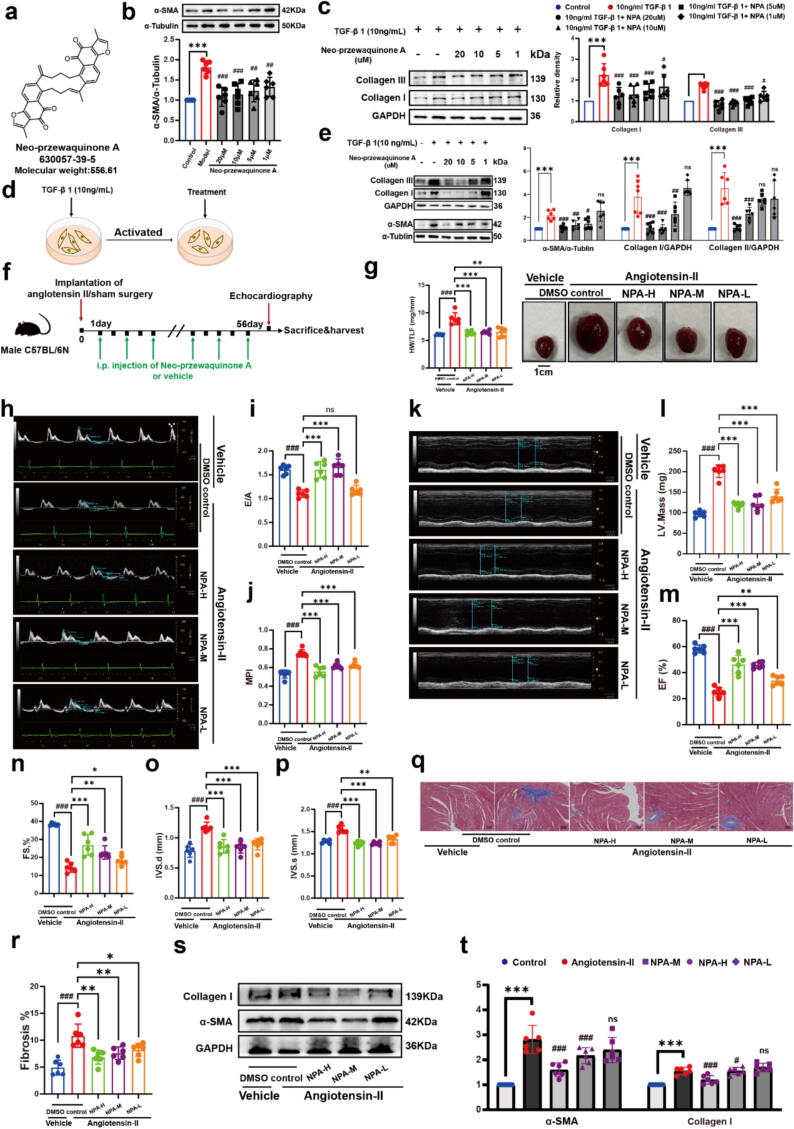
**a.** The structure of the NPA. **b.** NPA significantly inhibited α-SMA increase in NRCFs. **c.** NPA significantly inhibited Collagen I and Collagen III increase in NRCFs. **d.** The NIH-3T3 cells were activated by 10 ng/mL TGF-β I, after treated with different concentrations of NPA. **e.** The immunoblot analysis of α-SMA, Collagen I and Collagen III in NRCFs (n = 6). **f.** NPA or dimethyl sulfoxide (DMSO) was injected intraperitoneally every other day for 8 consecutive weeks starting after the implantation of minipumps filled with angiotensin II (Ang II). **g.** Whole heart images and heart weight/tibia length ratio (HW/TL) of Sham, Ang II, and Ang II mice treated with NPA, n = 6 for each group. (**h–i**) Representative pulsed-wave (PW) Doppler and tissue Doppler images from each group. (**k–p**) Echocardiography assessments of cardiac function were performed in different groups. The calculation of cardiac function included: left ventricular (LV) mass index, interventricular septal thickness at systole and diastole (IVS; s, IVS; d), n = 6 for each group. (**q.** and **r.**) Sections of hearts stained with Masson’s trichrome to detect fibrosis (blue) and quantification of cardiac fibrosis in different groups of mice, n = 3 for each group. For each slice, 3 fields of view were randomly selected for analysis, Scale bar: 100 μm. (**s.** and **t.**) Protein levels of fibrosis indices (α-SMA and Collagen-I) were determined by performing western blot analysis, n = 6 for each group. All data were analyzed using one-way ANOVA and were expressed as means ± SD, *P < 0.05, **P < 0.01, ***P < 0.001 versus Sham group, ^#^*P* < 0.05, ^##^*P* < 0.01, ^###^*P* < 0.001 versus Ang-II group.n s, statistically not significant.

By analyzing the 105 hit compounds derived from TCMs, 58 had not been previously reported for anti-fibrosis (Fig. S11a, Supporting Information). Some hits, such as Salvanic acid B, have been reported to treat myocardial fibrosis by regulating the TGF-β signaling pathway [45]. We paid particular attention to Neo-Przewaquinone A (NPA, Fig. 3a), a compound derived from *Salvia Linn* − *Salvia miltiorrhiza* Bge, which is commonly used in TCM for the treatment of cardiovascular disease [46]. Several studies have investigated the active ingredients in *Salvia miltiorrhiza* Bge for treating cardiac fibrosis [47], [48].

However, NPA has not yet been reported to exhibit anti-fibrotic effects. Besides, there is no FDA-approved drug that specifically targets cardiac fibrosis [49]. Therefore, we plan to use a cardiac fibrosis model to further explore NPA's potential for treating cardiac fibrosis.

### Anti-Cardiac fibrosis and cardiomyocyte toxicity of NPA

One of the identified hits, NPA (Fig. 3a), derived from *Salvia miltiorrhiza* Bge, was validated in a cardiac fibrosis model. It has not been previously reported for its ant-fibrotic effects [47], [48]. However, our results strongly support its preventive and therapeutic potential in cardiac fibrosis (Fig. 3, Fig. S12, Supporting Information). Primary neonatal rat cardiac fibroblasts (NRCFs) were stimulated with 10 ng/mL TGF-*β* I for 24 h, which significantly elevated α-SMA protein levels. Co-treatment with varying concentrations of NPA effectively inhibited the TGF-β I-induced increase in α-SMA expression (Fig. 3b). Additionally, NPA treatment restored the expression of Collagen I and Collagen III, which were upregulated by 10 ng/mL TGF-β I to near-normal levels (Fig. 3c). Interestingly, we found that NPA, derived from *Salvia miltiorrhiza* Bge, is a dimer of two compounds—Tanshinone I and Tanshinone IIA (Fig. S11b, Supporting Information). In Fig. S11c, the results showed that NPA exhibited similar anti-fibrotic effects to these two compounds by inhibiting α-SMA expression. Moreover, we compared the toxicity of these three compounds in myocardial cell lines (H9c2 cells). The results demonstrated that NPA caused less damage to cardiomyocytes than Tanshinone IIA (Fig. S11d, Supporting Information), making NPA a potentially safer option for therapeutic use. Notably, NPA has not been previously reported as a compound with lower toxicity than the other two compounds, while also showing a significant therapeutic effect in mitigating abnormal proliferation and changes in NRCFs (Fig. S11d, Supporting Information).

To further evaluate the therapeutic potential of NPA, protein levels of classical markers were analyzed after stimulation with 10 ng/mL TGF-β I and subsequent treatment with varying concentrations of NPA (Fig. 3d). As shown in Fig. 3e, NPA treatment led to a significant reduction in the levels of α-SMA, Collagen I, and Collagen III, which were otherwise elevated by TGF-β I stimulation. These findings suggest that NPA not only modulates key markers of fibrosis and induces cell morphology in cardiac fibroblasts, offering a promising new direction for the treatment and potential reversal of fibrosis.

### NPA reverses fibrosis in Angiotensin II (Ang II)–induced cardiac remodeling

To determine whether NPA can prevent or reverse the development of fibrosis *in vivo*, C57BL/6 N mice were subcutaneously injected with Ang II II for 8 weeks, while NPA (dissolved in 0.1 % dimethyl sulfoxide in saline) or vehicle (control) was administered intraperitoneally every other day. Cardiac fibrosis progression was assessed through histological analysis and echocardiographic evaluation of cardiac function. Although Ang II infusion significantly reduced body weight, NPA did not prevent this weight loss (Fig. S12a, Supporting Information). Ang II also induced significant cardiac hypertrophy, as indicated by an increased heart weight to the tibia length ratio (HW/TL) compared to the control group (no Ang II treatment). Treatment with different concentrations of NPA significantly reversed Ang II-induced changes in HW/TL (Fig. 3g).

In hypertensive mice, fibrosis development is associated with diastolic dysfunction, which can progress to systolic dysfunction and closely correlates with pathological remodeling seen in human myocardial fibrosis [50]. To assess the effect of NPA on diastolic function, tissue Doppler signals and mitral inflow were analyzed (Fig. 3h–p). Echocardiographic results revealed signs of diastolic dysfunction and impaired relaxation after 8 weeks of Ang II stimulation (Fig. 3h–p). NPA treatment improved several myocardial function parameters, including the myocardial performance index (MPI) and the E/A ratio, although these improvements were of borderline significance (Fig. 3i,j). Additionally, NPA treatment enhanced several echocardiographic parameters related to myocardial function, such as left ventricular mass (LV mass, Fig. 3i), left ventricular fractional shortening (FS%), and left ventricular ejection fraction (EF%). NPA also increased left ventricular end-diastolic and end-systolic volumes (LV Vol; d, LV Vol; s) compared to the Ang II infusion group (Fig. 3k–p).

Histological analysis using Masson staining demonstrated that NPA significantly reduced collagen deposition in myocardial tissue (Fig. 3q,r). Ang II-Induced fibrosis was accompanied by elevated TGF-β I expression and increased interstitial fibrosis (Fig. S12b and c, Supporting Information), along with upregulated α-SMA and collagen-I expression in heart tissue. NPA administration at different concentrations significantly inhibited these changes (Fig. 3s and t). NPA did not significantly affect Ang-II-induced myocardial hypertrophy (Fig. S12d, Supporting Information).

Regarding safety and tolerability, NPA treatment did not cause any signs of kidney and liver damage, as assessed by plasma markers (Fig. S12e–h, Supporting Information). Additionally, morphological analysis of kidney and liver tissues revealed no adverse effects following NPA administration (Fig. S12i, Supporting Information). To further investigate the distribution of NPA in vivo, liquid chromatography (LC) and quadrupole Ion trap mass spectrometer (QTRAP −MS) were used to analyze the blood and tissue distribution of NPA (Fig. S13a–e, Supporting Information). These *in vivo* results demonstrate that NPA has therapeutic potential for treating myocardial fibrosis and improving heart function, offering a promising new strategy for future myocardial fibrosis therapies.

### NPA as a novel inhibitor: Activation of the PI3K/AKT pathway via TGF-β receptor I and its effects on cell morphology during fibrosis

Our results have demonstrated the anti-fibrosis effects of NPA through morphological studies of fibrosis stimulated by TGF-β I, combined with unbiased machine learning. To determine whether NPA directly interferes with TGF-β receptor I (ALK5), SPR technology was employed to assess the binding affinity between the receptor protein and NPA.

**Fig. 4:**
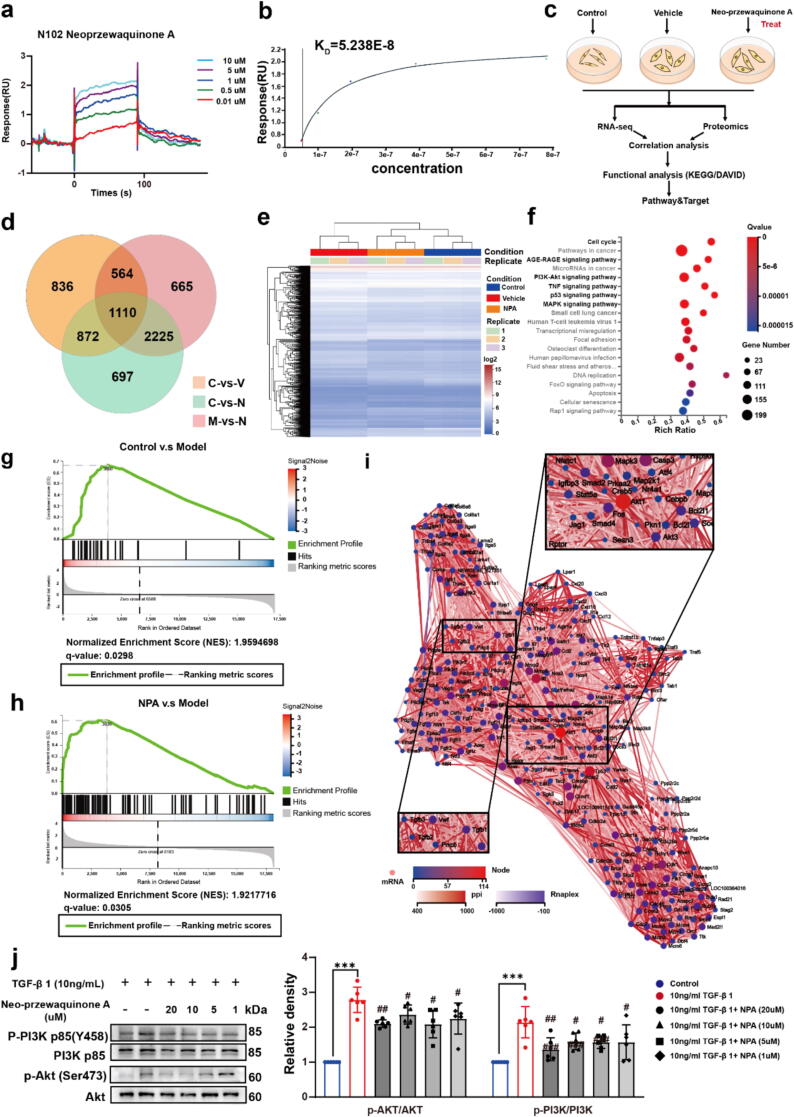
**(a.** and **b.)** Affinity between NPA and TGF-β receptor I. A series of concentrations (48–780 nM) of NPA were tested to obtain the affinity between NPA and TGF-β receptor by kinetic analysis. The KD value of the interaction between NPA and TGF-*β* receptor I was determined to be 5.38 × 10^−8^ M; **c.** NPA treated-NRCFs and control NRCFs were collected for RNA-seq analysis. (**d.** and **e.**) The differentially expressed genes (DEGs) were analysis. (**f**) The most significantly enriched Kyoto Encyclopedia of Genes and Genomes (KEGG) pathways. (**g.** and **h.**) GSEA of NPA-Treatment group showing a negative enrichment for profibrotic gene signatures. **i.** Through the interaction analysis of the related protein–protein interaction interface (PPI), Akt1 is highly enriched. **j.** PI3K/AKT protein and its Phosphorylated protein level in different groups. The results are expressed as the mean ± SD; *P < 0.05, **P < 0.01, ***P < 0.001, ^#^*P* < 0.05, ^##^*P* < 0.01, ^###^*P* < 0.001. ns, statistically not significant.

The CM5 sensor chip was prepared with TGF-β receptor I protein. The optimal isoelectric point for TGF-β receptor I was determined using Acetate acid at various pH levels, with pH 4.5 provided the highest immobilization levels (Fig. S14a, Supporting Information). The protein fixation level for channel-2 was 8538.70 RU (Fig. S14b, Supporting Information). NPA was diluted in water with 0.01 % DMSO to prepare a concentration series (0.01-10 *μ*M). The binding affinity of NPA to TGF-β receptor I was assessed using Biacore T200 Evaluation Software 3.0 (Fig. 4a). The *K_D_* value for the interactions between NPA, Tanshinone IIA, Tanshinone I and TGF-β receptor were 5.38 × 10^−8^ M, 3.47 × 10^−6^ M and 4.80 × 10^−7^ M, respectively (Fig. 4b, Fig. S14c and d, Supporting Information). These results indicate that NPA binds to TGF-β receptor I, which may contribute to its anti-fibrotic effects.

To explore how NPA affects the rapid proliferation of NRCFs, RNA-seq analysis was conducted on both NPA-treated and untreated NRCFs (Fig. 4c). A total of 1,110 differentially expressed genes (DEGs) were identified (Fig. 4d). mRNA libraries from untreated and NPA-treated NRCFs were sequenced, and gene ontology (GO) enrichment analysis revealed significant enrichment in biological processes related to cell proliferation and translation regulator activity (Fig. S14e, Supporting Information). Kyoto Encyclopedia of Genes and Genomes (KEGG) pathway analysis highlighted several enriched pathways, including cell cycle, AGE-RAGE signaling pathway, and PI3K/AKT signaling pathway (Fig. 4e and f). Next, RNA sequencing analysis was performed to compare global gene expression profiles in NRCFs treated with NPA, control, or model (10 ng/mL TGF-β I). Gene set enrichment analysis (GSEA) showed that TGF-β I upregulated genes in the PI3K/AKT signaling pathway, while NPA specifically downregulated genes within this pathway (Fig. S15a and b, Supporting Information). KEGG analysis confirmed significantly enrichment of the PI3K/AKT signaling pathway following NPA treatment (Fig. 4f). Protein-protein interaction (PPI) analysis further revealed that *Akt1* in the PI3K/AKT pathway, along with other TGF-β-related proteins, were highly enriched. (Fig. 4g).

**Fig. 5:**
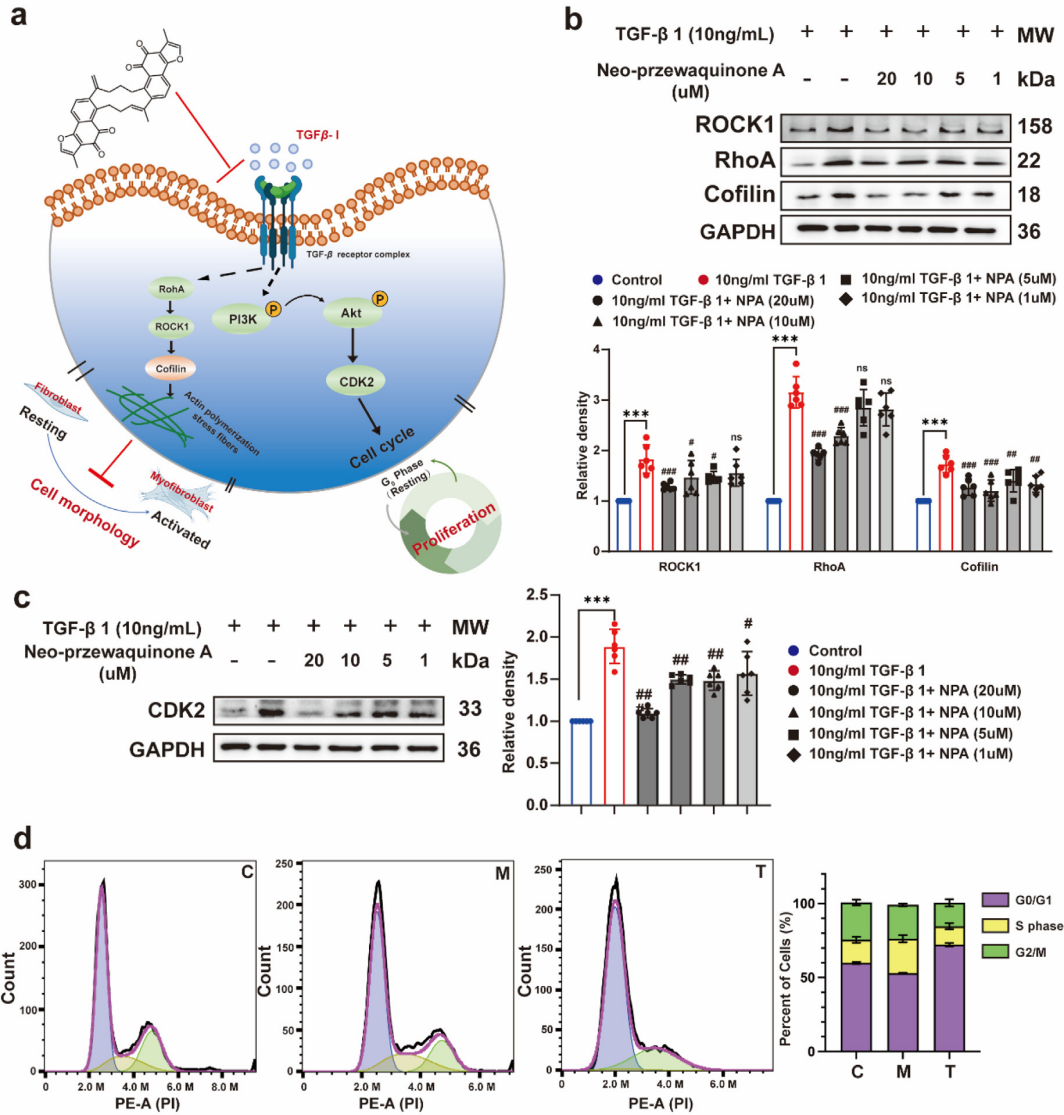
**a.** NPA affects the cytoskeleton through RhoA/ROCK1/Cofilin pathways and the cell cycle through CDK2. **b.** RhoA/ROCK1/Cofilin protein level in each group. **c.** CDK2 protein level in each group. **d.** Cell cycle analysis of control, 10 ng/mL TGF-β I and 10 ng/mL TGF-β I + NPA (10 μM). The results are expressed as the mean ± SD; *P < 0.05, **P < 0.01, ***P < 0.001, ^#^*P* < 0.05, ^##^*P* < 0.01, ^###^*P* < 0.001. ns, statistically not significant.

Immunoblotting confirmed that NPA-treated NRCFs exhibited reduced levels of phosphorylated AKT (p-AKT) and phosphorylated PI3K (p-PI3K) (Fig. 4h). Additionally, NPA treatment decreased levels of RhoA/ROCK1/Cofilin proteins, which are key regulators of cytoskeleton formation [51], consistent with our previous findings in NIH-3T3 cells using the *deep-DPC* screening method (Fig. 5a and b, Supporting Information).

Furthermore, KEGG analysis showed significant enrichment of the cell cycle signaling pathway (Fig. S15c, Supporting Information), suggesting that NPA inhibits fibrosis by delaying cell cycle progression *via* cyclin‐dependent kinase 2 (CDK2). Under TGF-β I stimulation, fibrocyte proliferation accelerates, worsening fibrosis [52]. Our results demonstrated that NPA affected CDK2 upregulation (Fig. 5c), and cell cycle analysis confirmed that NPA induced G1/S phase cell cycle arrest (Fig. 5d).

Transcriptome analysis revealed that NPA affects the PI3K/AKT pathway and modulates CDK2 expression *via* TGF-β receptor I, leading to cell cycle arrest and a reduction in fibrocyte proliferation (Fig. 4). Prior studies have demonstrated the critical role of CDK2 in cardiac fibrosis [52]. Additionally, NPA influences the RhoA/ROCK1/Cofilin pathway, regulating cytoskeletal morphology to maintain the original cell structure (Fig. 5). This approach may reveal molecular regulatory mechanism and open new avenues for anti-fibrotic drug discovery.

## Conclusion

We propose a novel strategy, *deep-DPC*, which utilizes deep learning to discriminate between the resting (fibroblast) and activated (myofibroblast) states by “controlling” cell morphology. Fibrosis is characterized by the excessive accumulation of extracellular matrix proteins, such as α-SMA, which leads to distinct cell morphology changes. Compared to traditional methods, DPC imaging offers the advantage of capturing real-time photographic recordings of cells without additional preparation. Moreover, DPC allows the dynamic observation of cellular morphology under compound influence over time, an ability lacking in conventional screening methods. Simultaneously, our data suggest that NPA has significant potential to reverse fibrosis in disease hearts, providing a new non-invasive, high-throughput method for drug discovery. As clinical therapy, *Salvia miltiorrhiza* Bge. has been widely used to treat fibrotic disease in millions of patients. However, the long-term safety of NPA (a compound from *Salvia miltiorrhiza* Bge.) for chronic antifibrotic therapy still needs to be comprehensively evaluated through large animal preclinical studies and clinical trials. Moreover, the establishment of *deep-DPC* provides an advanced method for DPC imaging in future drug screening and offers insights into cellular morphological mechanisms in disease. This approach holds promise for fibrosis and other diseases involving cellular morphology and matrix composition changes, such as cardiovascular diseases, aging, and cancer.

## Compliance with Ethics Requirements

All animal procedures were performed conforming to the National Institutes of Health guidelines and approved by the Institutional Animal Care and Use Committee of China Pharmaceutical University (2022–12-055).

## Authors’ contributions

Xu-dong Xing and Xiang-yu Yan performed experiments and analyzed data. Xu-dong Xing, Yang Liu, Yan-wei Tan, Yi-xin Cui, Chun-ling Feng, Yu-ru Cai and Han-lin Dai, supervised and analyzed in *vivo* experiments. Ping Zhou and Hui-ying Wang provided reagents to help design experiments. Writing–Original Draft, Xu-dong Xing, Wen Gao and Yi-xin Cui. The principal investigators, Ping Li and Hua Yang conceived the scientific ideas, oversaw the project, designed the experiments, and refined the manuscript. All authors discussed the results.

## Declaration of competing interest

The authors declare that they have no known competing financial interests or personal relationships that could have appeared to influence the work reported in this paper.
