# Supplementary material for: Deep-DPC: Deep learning-assisted label-free temporal imaging discovery of anti-fibrotic compounds by controlling cell morphology
Source: J Adv Res. 2025 Feb 24;78:703–16. doi: 10.1016/j.jare.2025.02.028 (PMC12685513; doi:10.1016/j.jare.2025.02.028)
Supplement: Supplementary Data 1 [file mmc1.docx]

**Supporting Information**

**Deep-DPC: Deep learning-assisted label-free temporal imaging discovery of anti-fibrotic compounds by controlling cell morphology**

**Authors**

Xu-dong Xing, Xiang-yu Yan, Yan-wei Tan, Yang Liu, Yi-xin Cui, Chun-ling Feng, Yu-ru Cai, Han-lin Dai, Wen Gao, Ping Zhou, Hui-ying Wang, Ping Li*, Hua Yang*

**Affiliations**

State Key Laboratory of Natural Medicines, School of Traditional Chinese Pharmacy, China Pharmaceutical University, No. 639 Longmian Dadao, Nanjing 211198, China

**
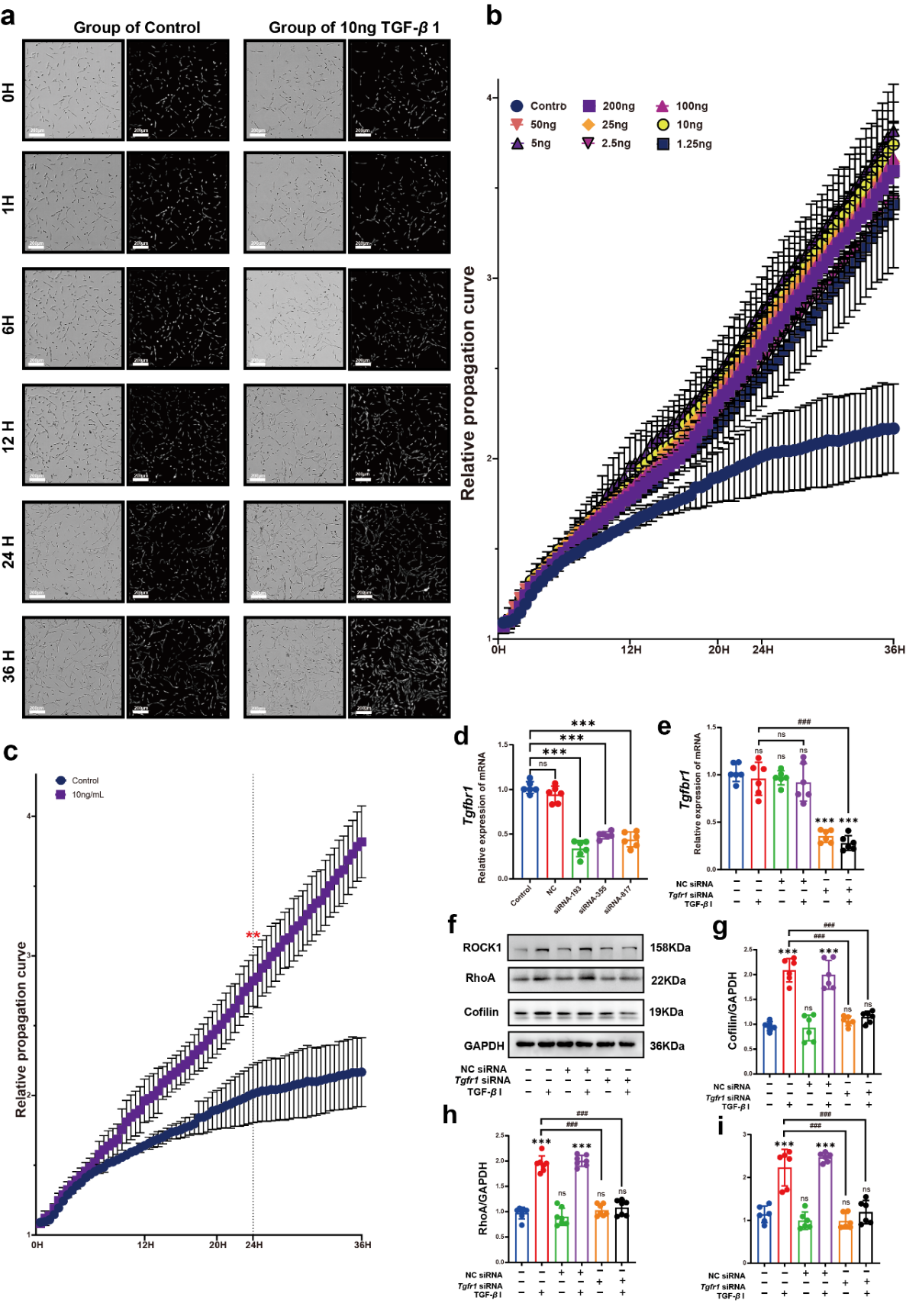
**

**Figure S1.** (a) The time sequence images and (b and c) relative proliferation curve was used to represent the amount of NIH-3T3 cell proliferation over 36 h. (d and e) The mRNA level of *Tgfbr1* was determined by quantitative PCR (n=6). (f-i) The immunoblot analysis of Cofilin, RhoA, and ROCK1 in NIH-3t3 cells in different groups. The results are expressed as the mean $\pm$ SD; **P* < 0.05, ***P* < 0.01, ****P* < 0.001, ^#^*P* < 0.05, ^##^*P* < 0.01, ^###^*P* < 0.001. ns, statistically not significant.


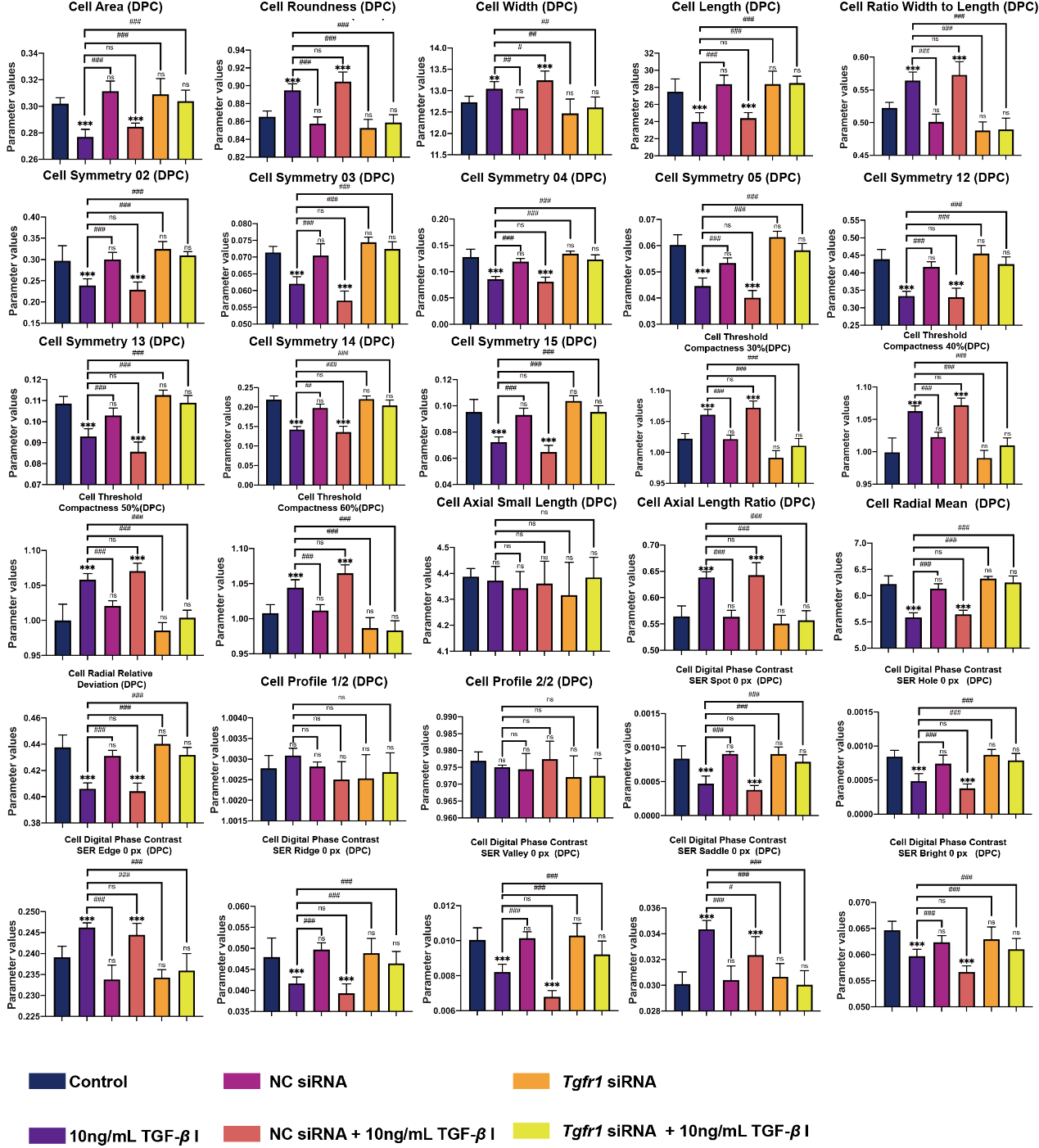


**Figure S2.** Analysis of parameters of DPC images from Harmony 4.9. The results are expressed as the mean $\pm$ SD; **P* < 0.05, ***P* < 0.01, ****P* < 0.001, ^#^*P* < 0.05, ^##^*P* < 0.01, ^###^*P* < 0.001. ns, statistically not significant.

**
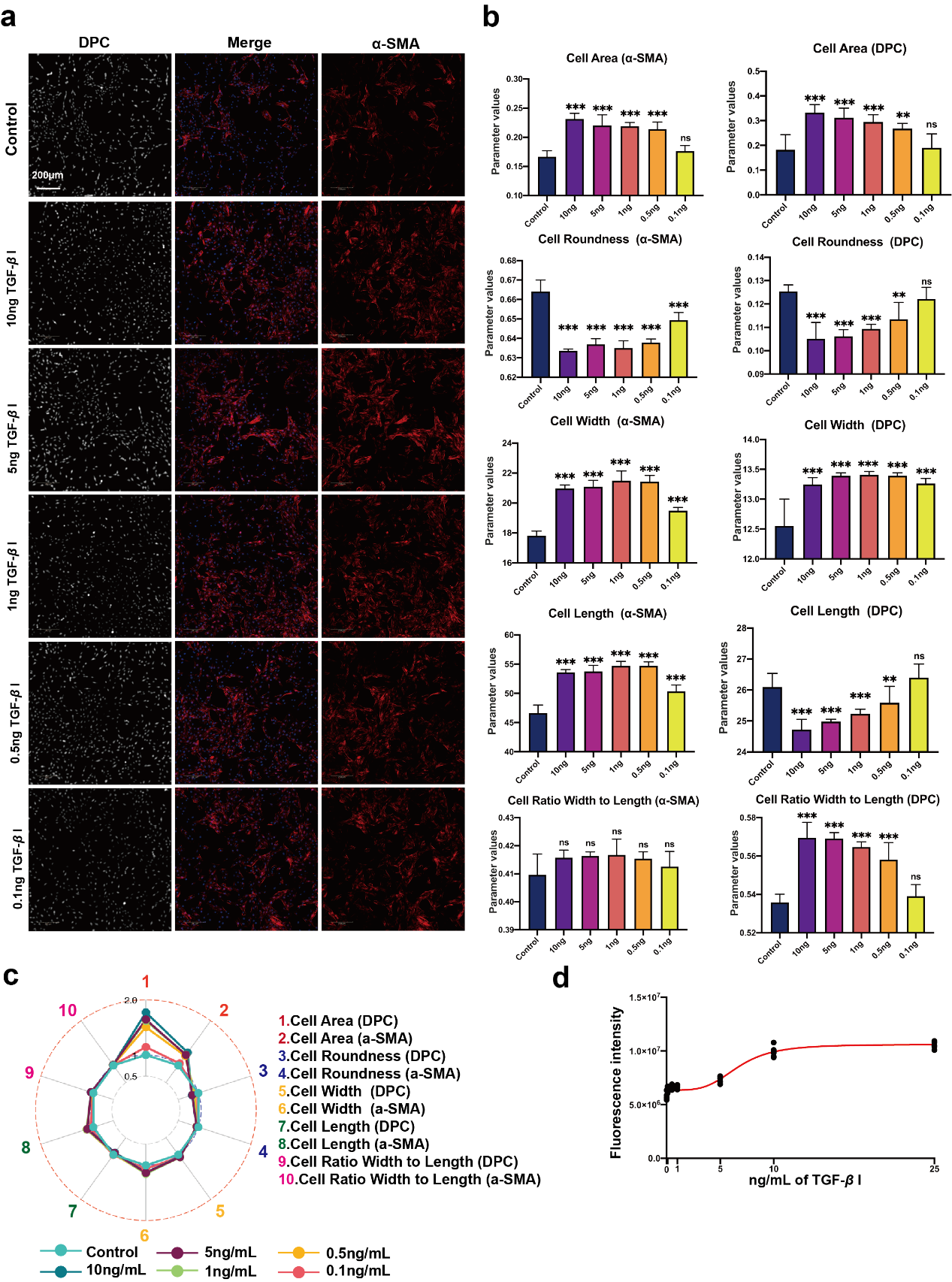
**

**Figure S3.** (a) The DPC image, the immunofluorescence images of α-SMA at different concentrations TGF-β I, Scale bar: 200 $\mu$m. (b) Analysis of parameters of DPC images and immunofluorescence images of α-SMA from Harmony 4.9. (c) Radar chart of multi-parametric morphology features of the DPC image and the immunofluorescence images of α-SMA image. (d) The fluorescence intensity of different concentrations TGF-β I. The results are expressed as the mean $\pm$ SD; **P* < 0.05, ***P* < 0.01, ****P* < 0.001, ^#^*P* < 0.05, ^##^*P* < 0.01, ^###^*P* < 0.001. ns, statistically not significant.

**
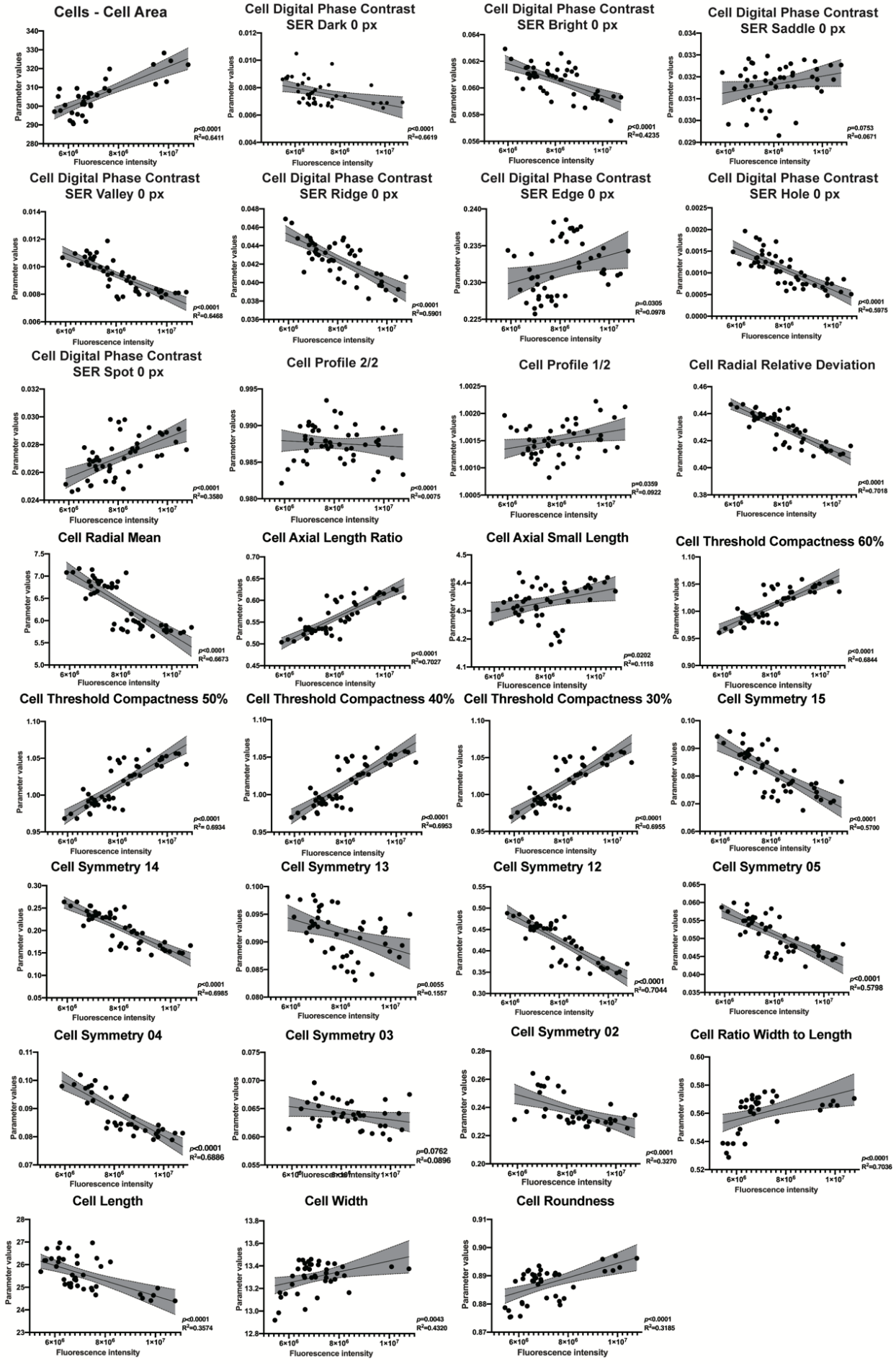
**

**Figure S4.** Analysis of parameters of DPC images and α-SMA fluorescence images by Harmony 4.9 software. The results are expressed as the mean $\pm$ SD; **P* < 0.05, ***P* < 0.01, ****P* < 0.001, ^#^*P* < 0.05, ^##^*P* < 0.01, ^###^*P* < 0.001. ns, statistically not significant.

**
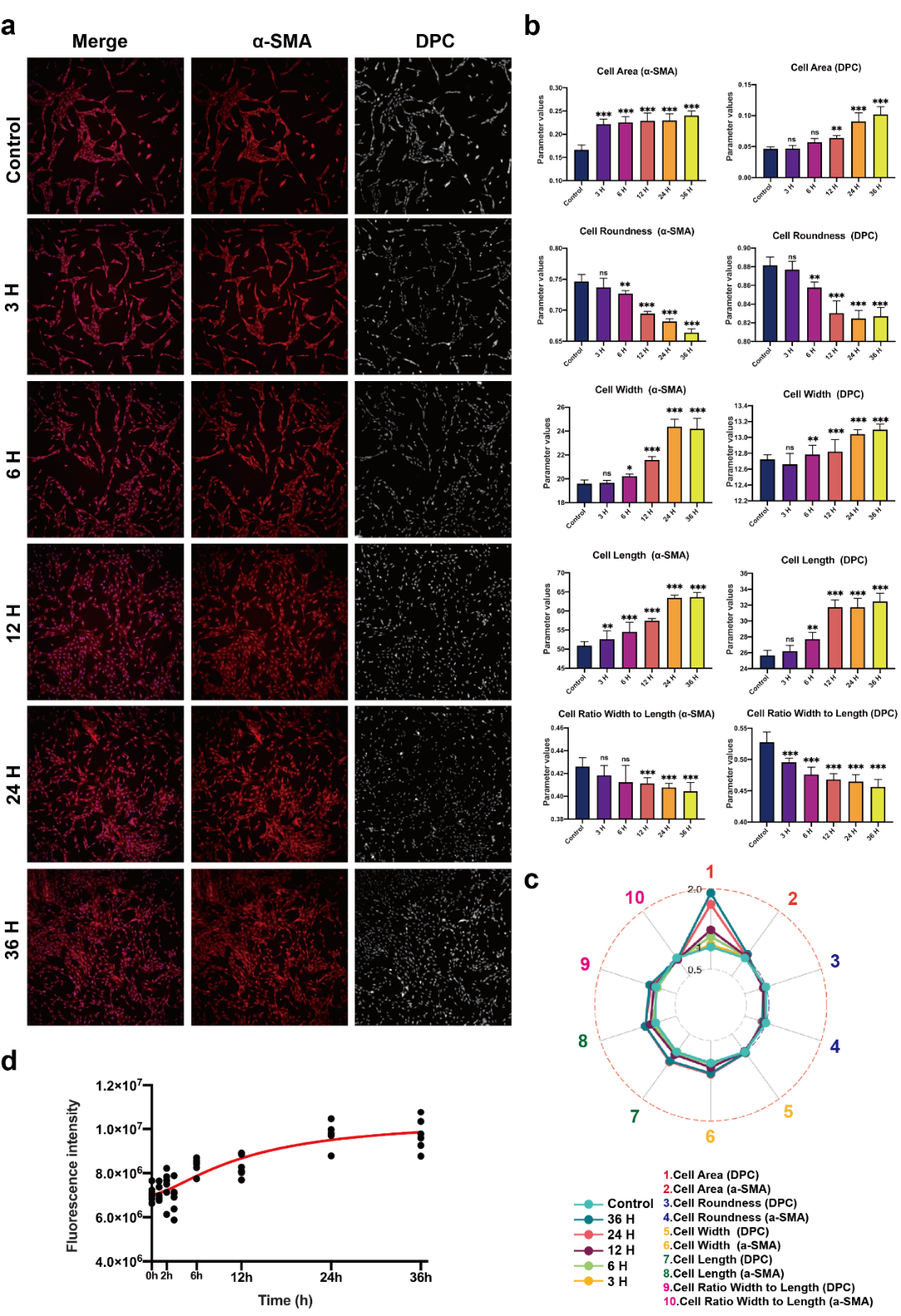
**

**Figure S5.** (a) DPC imaging and α-SMA fluorescence imaging at different times in the same concentration of TGF-β I, Scale bar: 200 $\mu$m. (b) Analysis of parameters of DPC images and immunofluorescence images of α-SMA from Harmony 4.9. (c) Radar chart of multi-parametric morphology features of the DPC image and the immunofluorescence images of α-SMA image. (d) The fluorescence intensity of 10ng/mL TGF-β I at different times. The results are expressed as the mean $\pm$ SD; **P* < 0.05, ***P* < 0.01, ****P* < 0.001, ^#^*P* < 0.05, ^##^*P* < 0.01, ^###^*P* < 0.001. ns, statistically not significant.

**
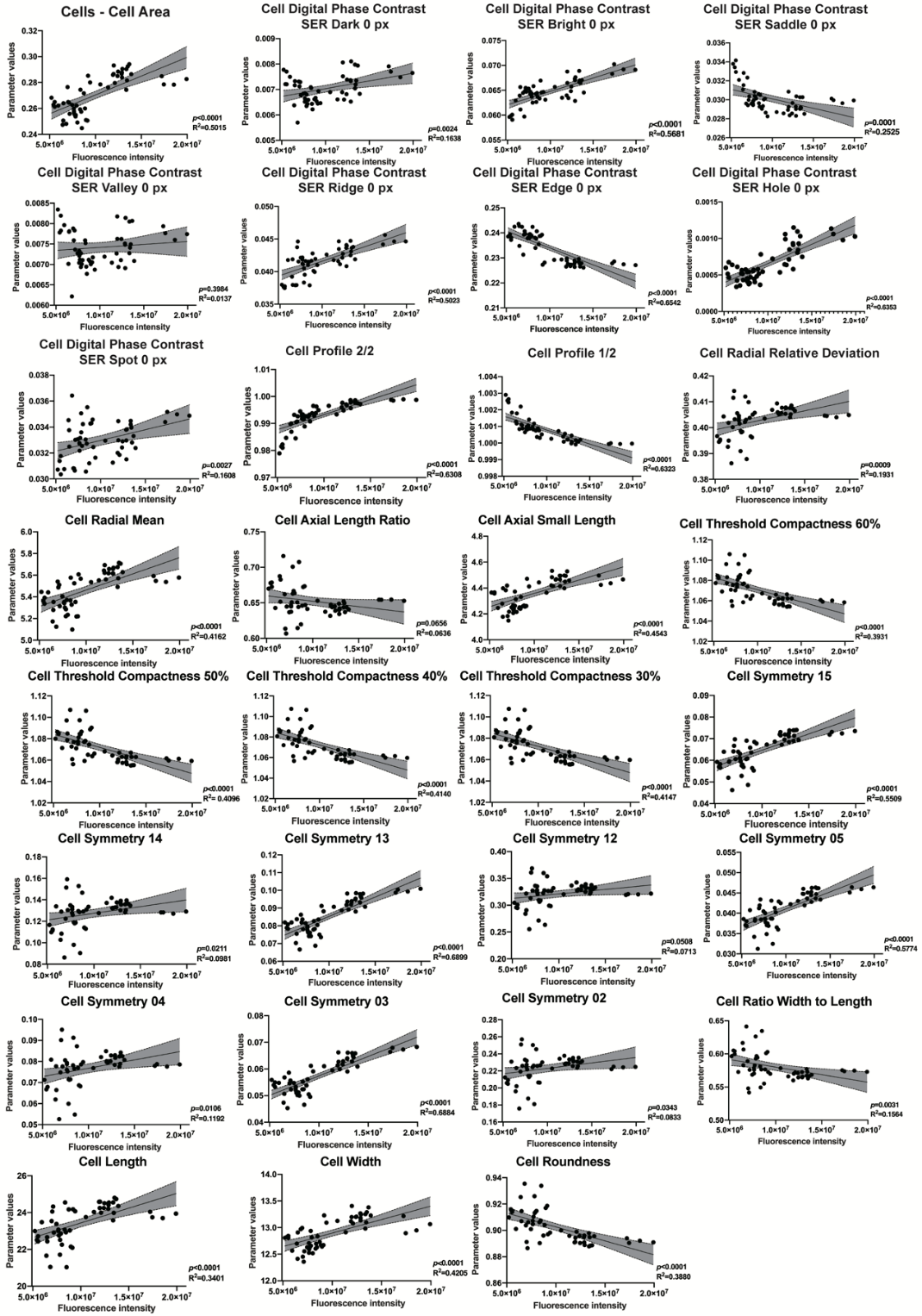
**

**Figure S6.** Analysis of parameters of DPC images and α-SMA fluorescence images by Harmony 4.9 software. The results are expressed as the mean $\pm$ SD; **P* < 0.05, ***P* < 0.01, ****P* < 0.001, ^#^*P* < 0.05, ^##^*P* < 0.01, ^###^*P* < 0.001. ns, statistically not significant.

**
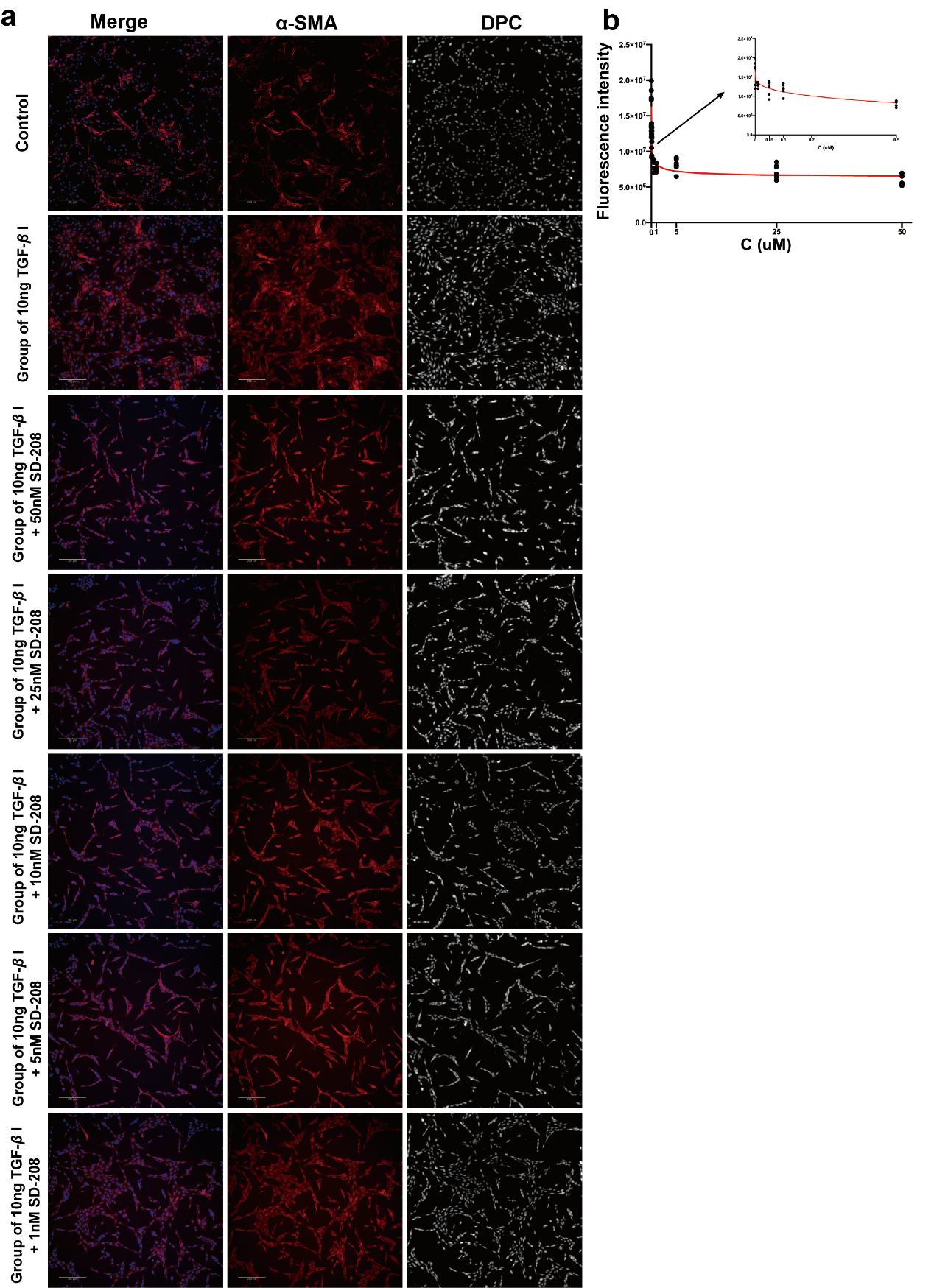
**

**Figure S7.** (a) The DPC and α-SMA fluorescence imaging by different concentrations SD-208 with 10ng/mL TGF-β I, Scale bar: 200 $\mu$m. (b) The fluorescence intensity of different concentrations SD-208 with 10ng/mL TGF-β I. The results are expressed as the mean $\pm$ SD; **P* < 0.05, ***P* < 0.01, ****P* < 0.001, ^#^*P* < 0.05, ^##^*P* < 0.01, ^###^*P* < 0.001. ns, statistically not significant.

**
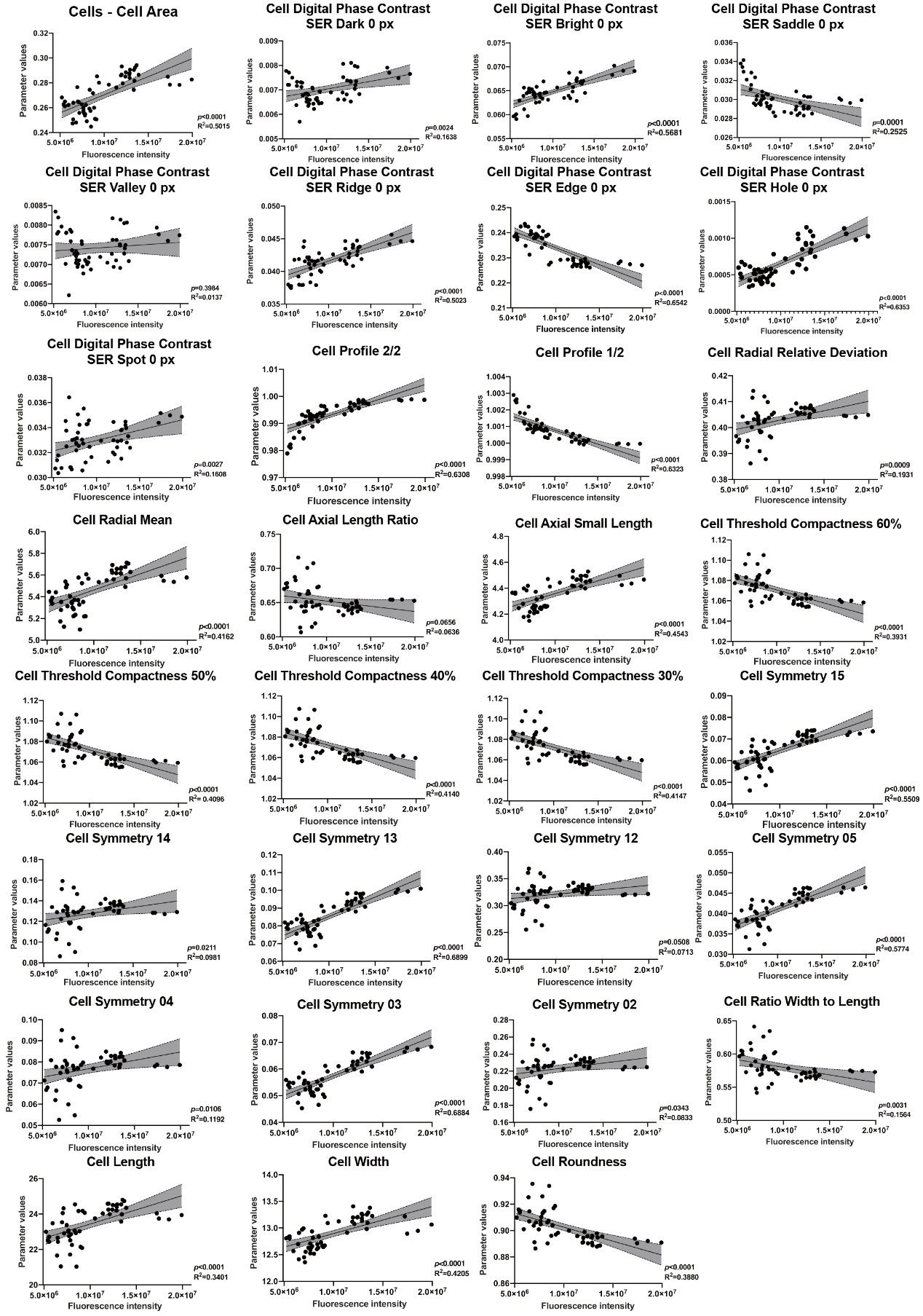
**

**Figure S8.** Analysis of parameters of DPC images and α-SMA fluorescence images by Harmony 4.9 software. The results are expressed as the mean $\pm$ SD; **P* < 0.05, ***P* < 0.01, ****P* < 0.001, ^#^*P* < 0.05, ^##^*P* < 0.01, ^###^*P* < 0.001. ns, statistically not significant.

**
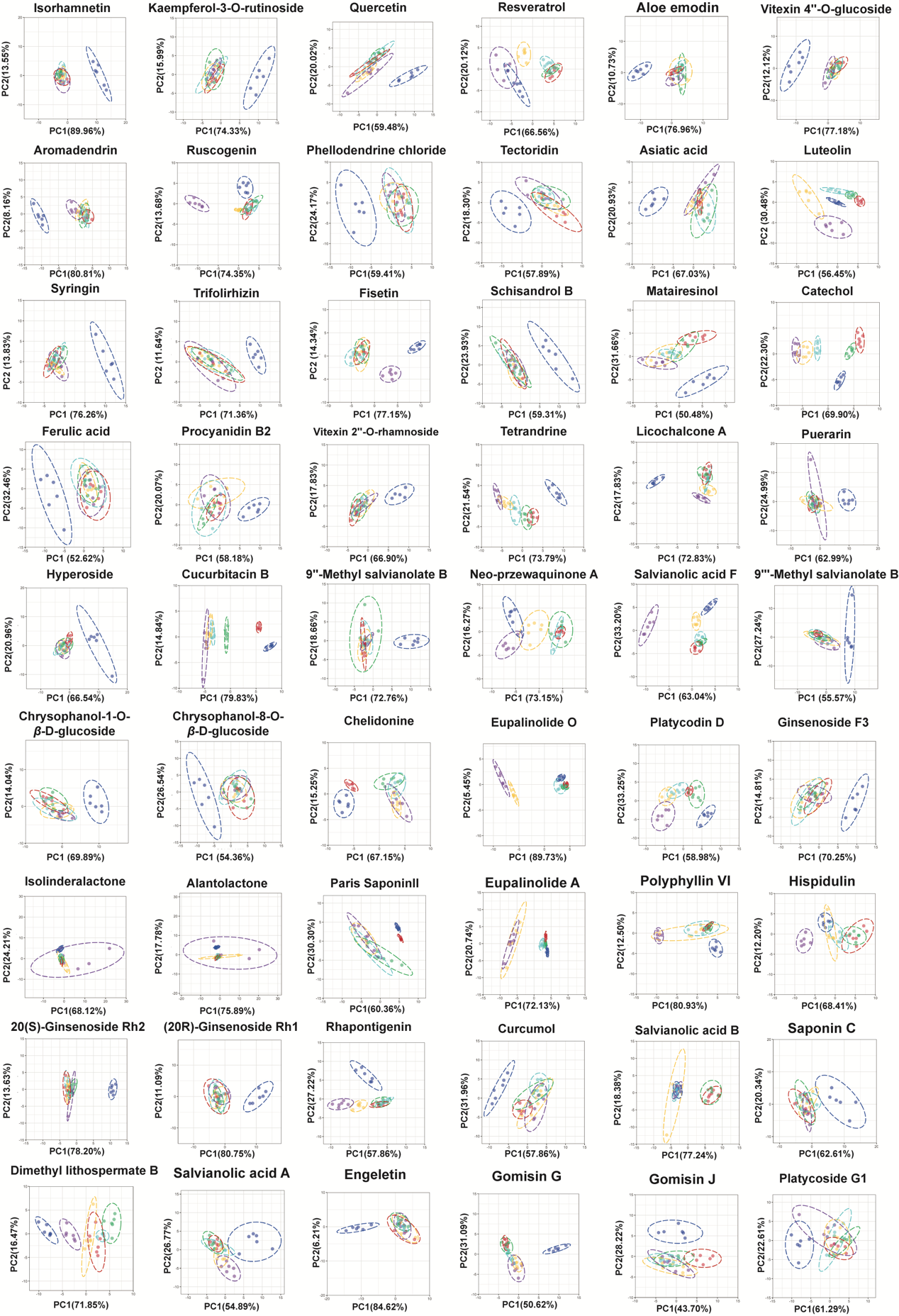
**

**
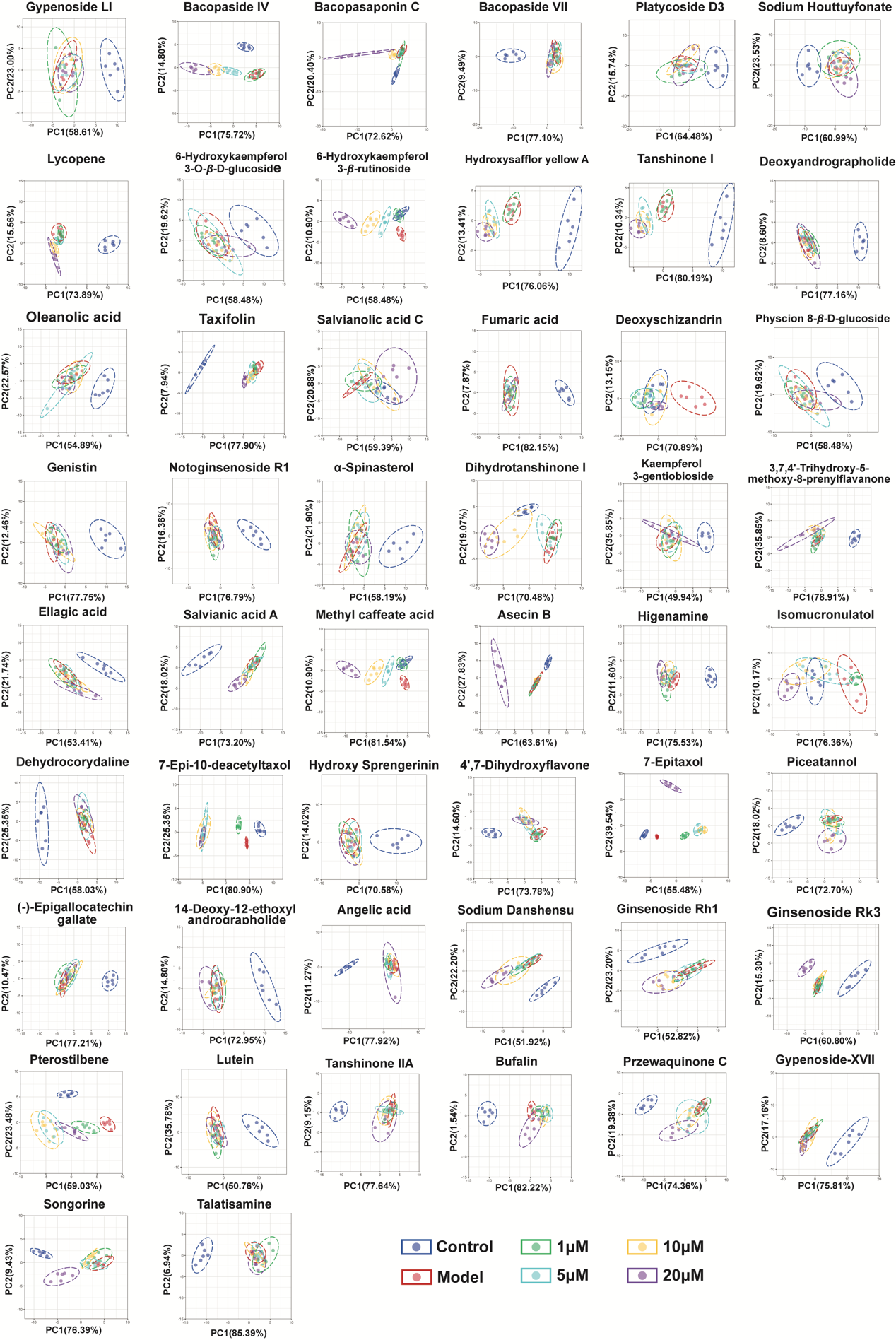
**

**Figure S9.** Principal component analysis (PCA) to perform latitudinal clustering analysis.

**
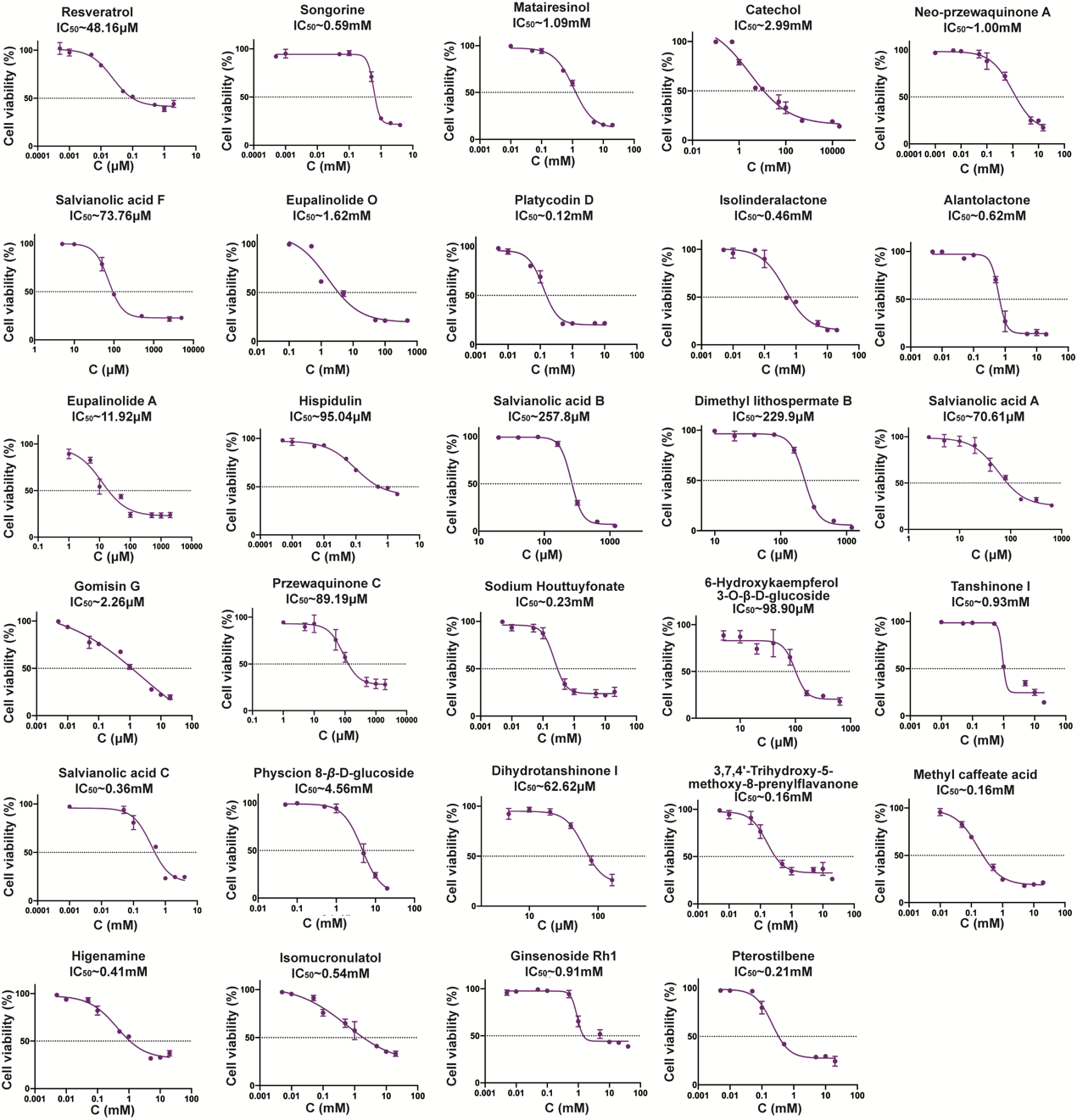
**

**Figure S10.** IC_50_ toxicity test on NIH-3T3 cells for these 29 hit compounds.

**
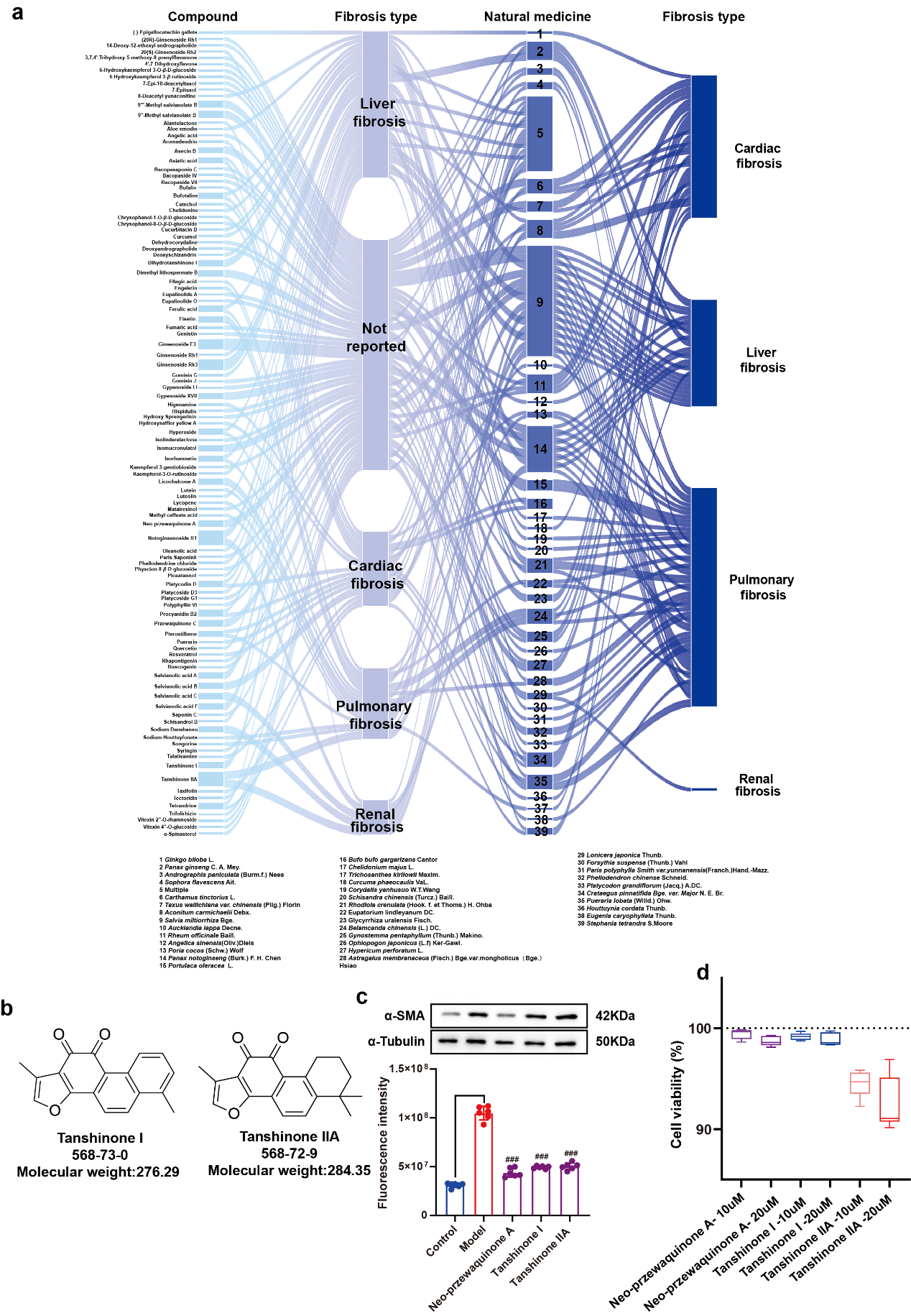
**

**Figure S11.** (a) Origin and Analysis of Natural Products. (b) The structure of the Tanshinone I and Tanshinone II A. (c) Three compounds significantly inhibited α-SMA increase in NRCFs. (d) Toxicity of three compounds to myocardial cell line (H9c2 cells).

**
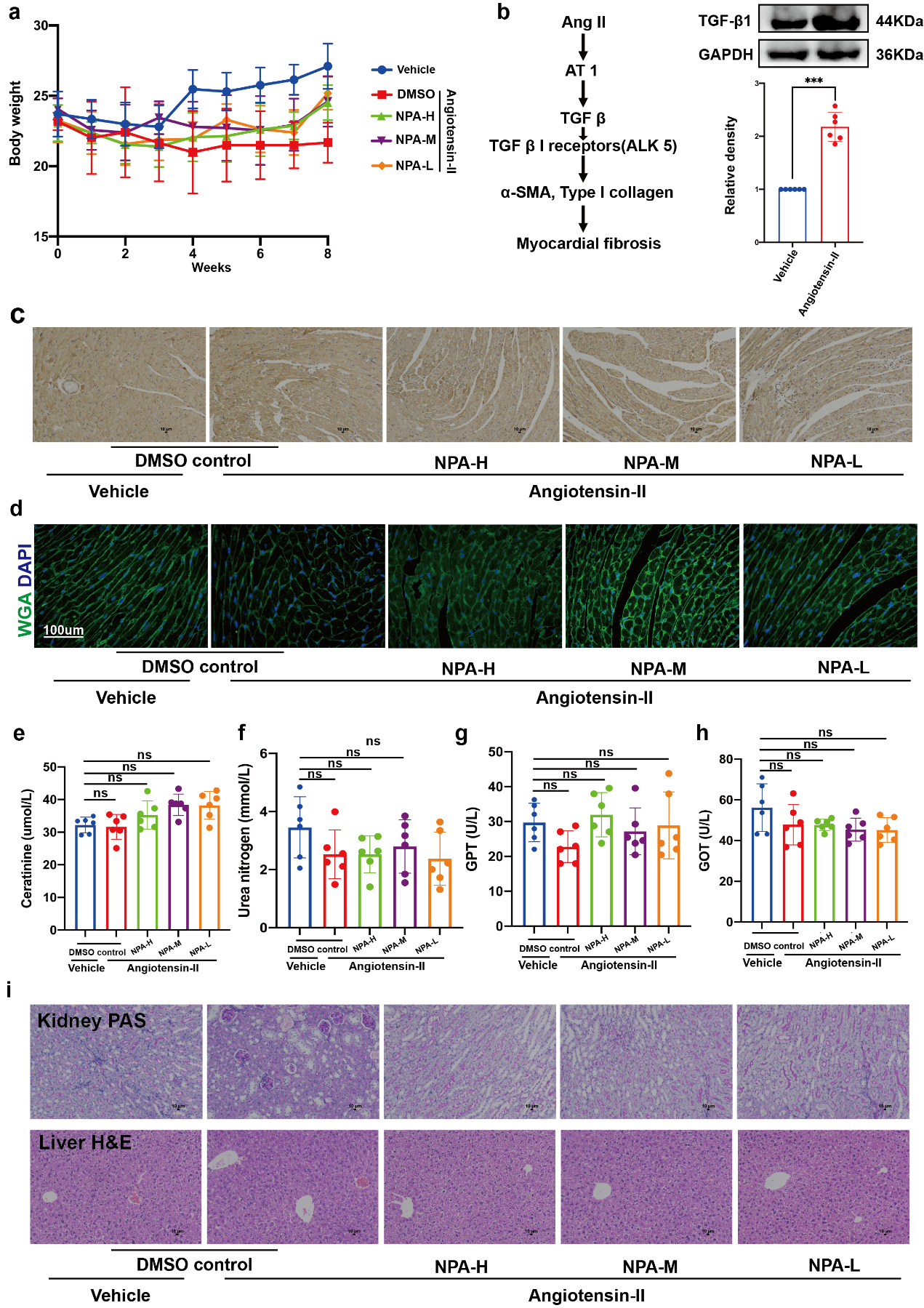
**

**Figure S12.** (a) The body weight in each group. (b and c) Ang II-induced elevated tissue TGF-β and then significantly accumulated interstitial space fibrosis. (d) Cardiomyocyte size is shown in representative images, scale bar: 100 μm.. (e-h) Overall, levels of markers of kidney (creatinine and urea) and liver (glutamate-oxaloacetate-transaminase, GOT and glutamate-pyruvate-transaminase, GPT) damage remain unchanged upon treatment of mice with NPA for 8 weeks. (i) PAS-staining of kidneys (upper panel) and H&E of livers (lower panel) of mice after 8 weeks of AngII-infusion and 8 weeks treatment with NPA, scale bar: 10 μm. The results are expressed as the mean $\pm$ SD; **P* < 0.05, ***P* < 0.01, ****P* < 0.001, ^#^*P* < 0.05, ^##^*P* < 0.01, ^###^*P* < 0.001. ns, statistically not significant.

**
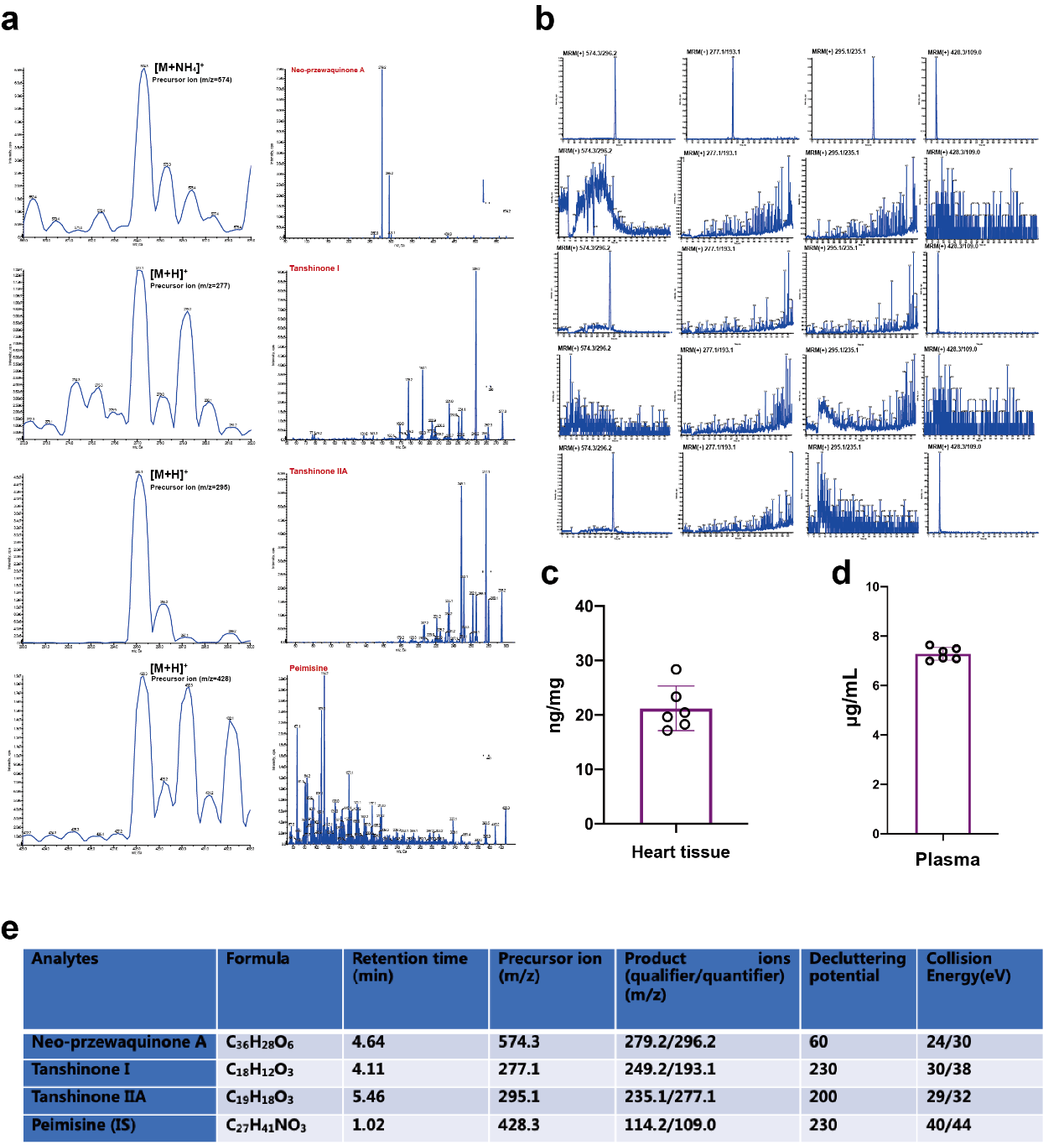
**

**Figure S13.** Quantification of Neo-Przewaquinone A by liquid chromatography (LC) and quadrupole Ion trap mass spectrometer (QTRAP -MS)

**
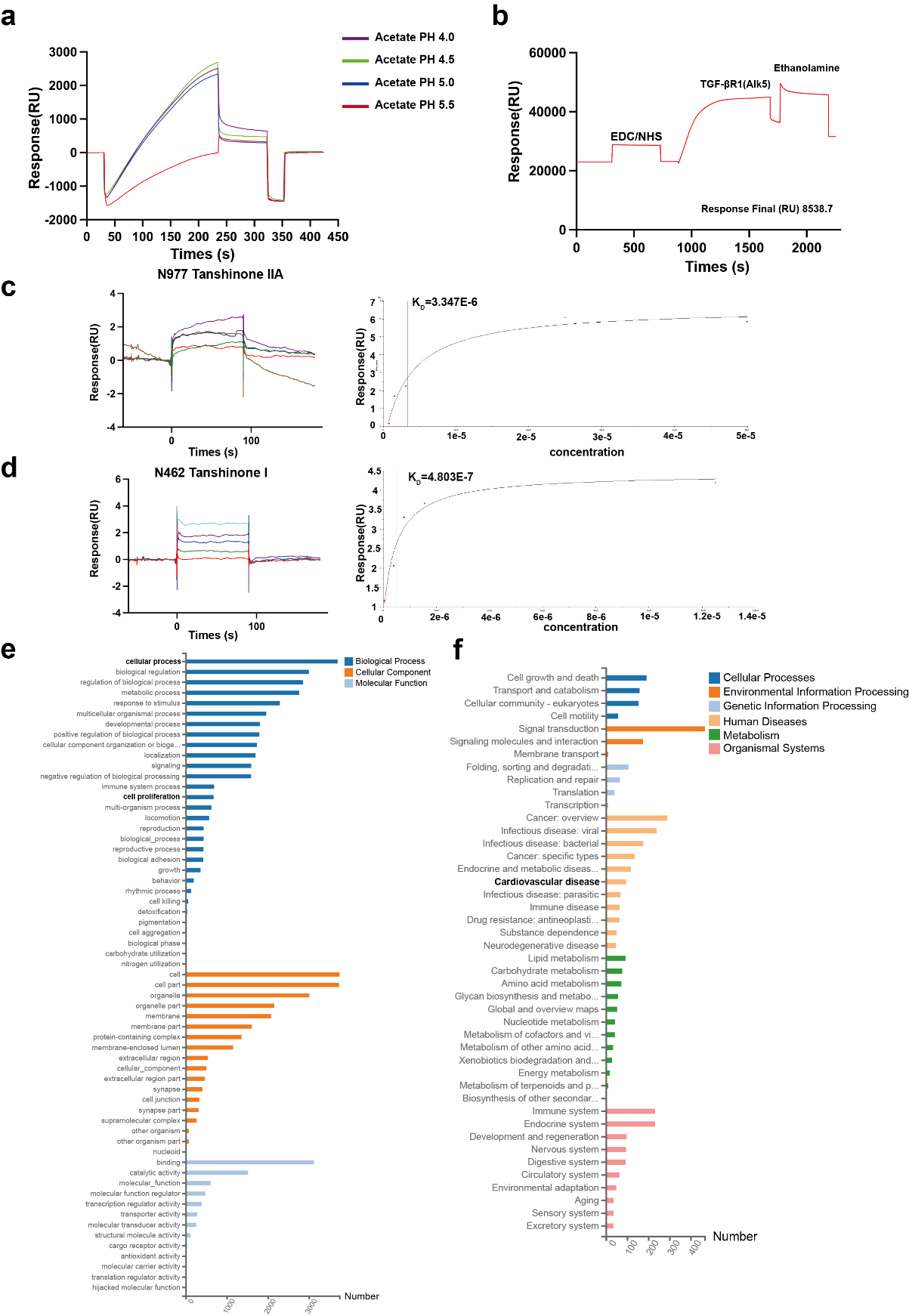
**

**Figure S14.** (a) Response of immobilization assay at different pH levels. (b) Amino coupling of TGF-β receptor with the wizard model. (c and d) Affinity between Tanshinone I, Tanshinone II and TGF-β receptor, respectively. The KD value of the interaction between Tanshinone I, Tanshinone II and TGF-β receptor was determined to be 3.47 × 10^−6^ M and 4.80 × 10^−7^ M, respectively. (e and f) Based on the GO enrichment analysis of the cis targeted genes of mRNA, Kyoto Encyclopedia of Genes and Genomes (KEGG). The results are expressed as the mean $\pm$ SD; **P* < 0.05, ***P* < 0.01, ****P* < 0.001, ^#^*P* < 0.05, ^##^*P* < 0.01, ^###^*P* < 0.001. ns, statistically not significant.

**
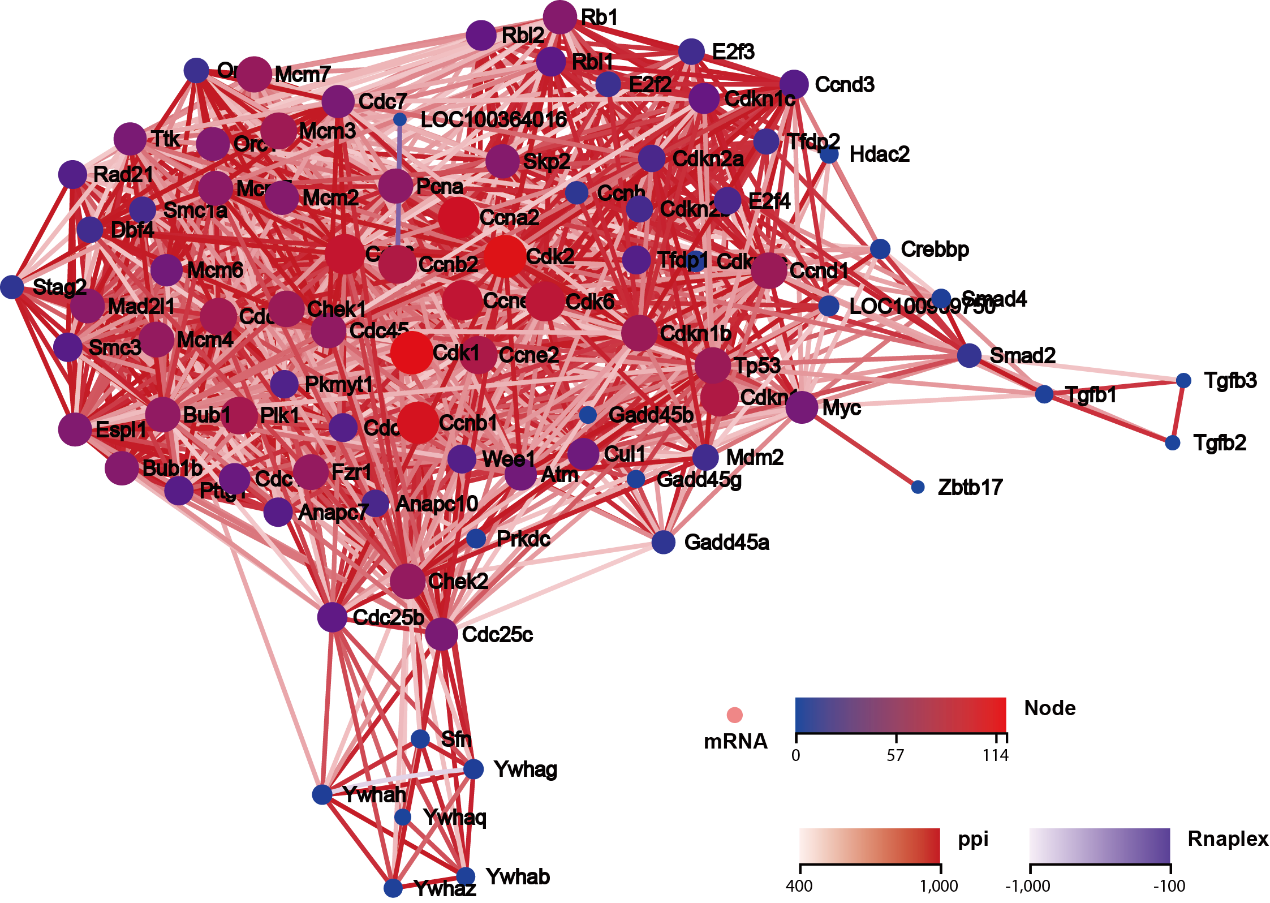
**

**Figure S15.** Through the interaction analysis of the related protein-protein interaction interface (PPI) in the cell cycle pathways, CDK2 is highly enriched.

**Supporting Information Table S1 The accuracy, precision, recall, F1 score comparison of all datasets**

| Model | Accuracy | Precision | Recall | F1 score |
| --- | --- | --- | --- | --- |
| Inception V4 | 0.922±0.03 | 0.932±0.05 | 0.922±0.03 | 0.927±0.07 |
| Inception-ResNet V2 | 0.881±0.03 | 0.884±0.03 | 0.880±0.05 | 0.882±0.06 |
| ResNet101 | 0.809±0.04 | 0.809±0.03 | 0.809±0.04 | 0.809±0.03 |
| VGG19 | 0.735±0.01 | 0.739±0.05 | 0.735±0.01 | 0.736±0.03 |

**Supporting Information Table S2.** The listed of siRNA sequence.

| Gene | siRNA |
| --- | --- |
| ***Tgfbr1*** | sense（5'-3'）：CCUCUGUACAAAGGAUAAUTT |
|  | antisense（5'-3'）：AUUAUCCUUUGUACAGAGGTT |

**Supporting Information Table S3.** 13 common perturbed DPC parameters.

| No. | Morphological parameter |
| --- | --- |
| 1 | Cell Area |
| 2 | Cell Digital Phase Contrast SER Bright 0 px |
| 3 | Cell Digital Phase Contrast SER Hole 0 px |
| 4 | Cell Digital Phase Contrast SER Ridge 0 px |
| 5 | Cell Length |
| 6 | Cell Radial Mean |
| 7 | Cell Roundness |
| 8 | Cell Symmetry 05 |
| 9 | Cell Symmetry 15 |
| 10 | Cell Threshold Compactness 30% |
| 11 | Cell Threshold Compactness 40% |
| 12 | Cell Threshold Compactness 50% |
| 13 | Cell Threshold Compactness 60% |

**Supporting Information Table S4.** The 105 hit compounds.

| No. | Name | CAS | Structure |
| --- | --- | --- | --- |
| 1 | Isorhamnetin | 480-19-3 |  |
| 2 | Kaempferol-3-O-rutinoside | 17650-84-9 |  |
| 3 | Resveratrol | [501-36-0](https://scifinder-n.cas.org/navigate/?appId=0b812ad8-214f-467c-9216-a005007c43bd&backKey=620c59d1845e2a1c58dafd37&backToPage=1&contentUri=substance%2Fpt%2F501360&key=620c59d1845e2a1c58dafd37&metricsOrdinal=1&metricsResultType=substance&ordinal=1&resultType=substance&resultView=DETAIL&sortBy=relevance&sortOrder=descending&state=searchDetail.substance&uiContext=365&uiSubContext=551&uriForDetails=substance%2Fpt%2F501360) |  |
| 4 | Aloe emodin | [481-72-1](https://scifinder-n.cas.org/navigate/?appId=ee41cf3e-f4aa-402e-a3f1-da33fb255628&backKey=620c5af1845e2a1c58daff62&clearSearch=true&contentUri=substance%2Fpt%2F481721&isFromAllResults=true&key=620c5af2845e2a1c58daff65&metricsOrdinal=1&metricsResultType=all&ordinal=1&resultType=substance&resultView=DETAIL&state=searchDetail.substance&suppressNavigation=true&uiContext=676&uiSubContext=551&uriForDetails=substance%2Fpt%2F481721) |  |
| 5 | Vitexin 4''-O-glucoside | 178468-00-3 |  |
| 6 | Aromadendrin | [480-20-6](https://scifinder-n.cas.org/navigate/?appId=ee41cf3e-f4aa-402e-a3f1-da33fb255628&backKey=620c5b6d845e2a1c58db006f&clearSearch=true&contentUri=substance%2Fpt%2F480206&isFromAllResults=true&key=620c5b6e845e2a1c58db0072&metricsOrdinal=1&metricsResultType=all&ordinal=1&resultType=substance&resultView=DETAIL&state=searchDetail.substance&suppressNavigation=true&uiContext=676&uiSubContext=551&uriForDetails=substance%2Fpt%2F480206) |  |
| 7 | Ruscogenin | [472-11-7](https://scifinder-n.cas.org/navigate/?appId=ee41cf3e-f4aa-402e-a3f1-da33fb255628&backKey=620c5b9c845e2a1c58db00c9&clearSearch=true&contentUri=substance%2Fpt%2F472117&isFromAllResults=true&key=620c5b9d845e2a1c58db00ca&metricsOrdinal=1&metricsResultType=all&ordinal=1&resultType=substance&resultView=DETAIL&state=searchDetail.substance&suppressNavigation=true&uiContext=676&uiSubContext=551&uriForDetails=substance%2Fpt%2F472117) |  |
| 8 | Phellodendrine chloride | 104112-82-5 |  |
| 9 | Tectoridin | [611-40-5](https://scifinder-n.cas.org/navigate/?appId=ee41cf3e-f4aa-402e-a3f1-da33fb255628&backKey=620c5bd2845e2a1c58db013b&clearSearch=true&contentUri=substance%2Fpt%2F611405&isFromAllResults=true&key=620c5bd3845e2a1c58db013f&metricsOrdinal=1&metricsResultType=all&ordinal=1&resultType=substance&resultView=DETAIL&state=searchDetail.substance&suppressNavigation=true&uiContext=676&uiSubContext=551&uriForDetails=substance%2Fpt%2F611405) |  |
| 10 | Asiatic acid | [464-92-6](https://scifinder-n.cas.org/navigate/?appId=ee41cf3e-f4aa-402e-a3f1-da33fb255628&backKey=620c5d2b845e2a1c58db0431&backToPage=1&contentUri=substance%2Fpt%2F464926&key=620c5d2b845e2a1c58db0431&metricsOrdinal=1&metricsResultType=substance&ordinal=1&resultType=substance&resultView=DETAIL&sortBy=relevance&sortOrder=descending&state=searchDetail.substance&uiContext=365&uiSubContext=551&uriForDetails=substance%2Fpt%2F464926) |  |
| 11 | Luteolin | [491-70-3](https://scifinder-n.cas.org/navigate/?appId=ee41cf3e-f4aa-402e-a3f1-da33fb255628&backKey=620c5d3f845e2a1c58db0465&backToPage=1&contentUri=substance%2Fpt%2F491703&key=620c5d3f845e2a1c58db0465&metricsOrdinal=1&metricsResultType=substance&ordinal=1&resultType=substance&resultView=DETAIL&sortBy=relevance&sortOrder=descending&state=searchDetail.substance&uiContext=365&uiSubContext=551&uriForDetails=substance%2Fpt%2F491703) |  |
| 12 | Syringin | [118-34-3](https://scifinder-n.cas.org/navigate/?appId=ee41cf3e-f4aa-402e-a3f1-da33fb255628&backKey=620c5d5e845e2a1c58db04a4&backToPage=1&contentUri=substance%2Fpt%2F118343&key=620c5d5e845e2a1c58db04a4&metricsOrdinal=1&metricsResultType=substance&ordinal=1&resultType=substance&resultView=DETAIL&sortBy=relevance&sortOrder=descending&state=searchDetail.substance&uiContext=365&uiSubContext=551&uriForDetails=substance%2Fpt%2F118343) |  |
| 13 | Trifolirhizin | [6807-83-6](https://scifinder-n.cas.org/navigate/?appId=ee41cf3e-f4aa-402e-a3f1-da33fb255628&backKey=620c5d98845e2a1c58db051a&backToPage=1&contentUri=substance%2Fpt%2F6807836&key=620c5d98845e2a1c58db051a&metricsOrdinal=1&metricsResultType=substance&ordinal=1&resultType=substance&resultView=DETAIL&sortBy=relevance&sortOrder=descending&state=searchDetail.substance&uiContext=365&uiSubContext=551&uriForDetails=substance%2Fpt%2F6807836) |  |
| 14 | Fisetin | [528-48-3](https://scifinder-n.cas.org/navigate/?appId=ee41cf3e-f4aa-402e-a3f1-da33fb255628&backKey=620c5dd6845e2a1c58db0596&backToPage=1&contentUri=substance%2Fpt%2F528483&key=620c5dd6845e2a1c58db0596&metricsOrdinal=1&metricsResultType=substance&ordinal=1&resultType=substance&resultView=DETAIL&sortBy=relevance&sortOrder=descending&state=searchDetail.substance&uiContext=365&uiSubContext=551&uriForDetails=substance%2Fpt%2F528483) |  |
| 15 | 3,7,4'-Trihydroxy-5-methoxy-8-prenylflavanone | 204935-85-3 |  |
| 16 | Matairesinol | [580-72-3](https://scifinder-n.cas.org/navigate/?appId=ee41cf3e-f4aa-402e-a3f1-da33fb255628&backKey=620c5dfa845e2a1c58db05de&backToPage=1&contentUri=substance%2Fpt%2F580723&key=620c5dfa845e2a1c58db05de&metricsOrdinal=1&metricsResultType=substance&ordinal=1&resultType=substance&resultView=DETAIL&sortBy=relevance&sortOrder=descending&state=searchDetail.substance&uiContext=365&uiSubContext=551&uriForDetails=substance%2Fpt%2F580723) |  |
| 17 | Catechol | [120-80-9](https://scifinder-n.cas.org/navigate/?appId=ee41cf3e-f4aa-402e-a3f1-da33fb255628&backKey=620c6b37845e2a1c58db21f1&backToPage=1&contentUri=substance%2Fpt%2F120809&key=620c6b37845e2a1c58db21f1&metricsOrdinal=1&metricsResultType=substance&ordinal=1&resultType=substance&resultView=DETAIL&sortBy=relevance&sortOrder=descending&state=searchDetail.substance&uiContext=365&uiSubContext=551&uriForDetails=substance%2Fpt%2F120809) |  |
| 18 | Ferulic acid | [1135-24-6](https://scifinder-n.cas.org/navigate/?appId=ee41cf3e-f4aa-402e-a3f1-da33fb255628&backKey=620c6b4b845e2a1c58db2212&backToPage=1&contentUri=substance%2Fpt%2F1135246&key=620c6b4b845e2a1c58db2212&metricsOrdinal=1&metricsResultType=substance&ordinal=1&resultType=substance&resultView=DETAIL&sortBy=relevance&sortOrder=descending&state=searchDetail.substance&uiContext=365&uiSubContext=551&uriForDetails=substance%2Fpt%2F1135246) |  |
| 19 | Procyanidin B2 | [29106-49-8](https://scifinder-n.cas.org/navigate/?appId=ee41cf3e-f4aa-402e-a3f1-da33fb255628&backKey=620c6b5c845e2a1c58db2232&backToPage=1&contentUri=substance%2Fpt%2F29106498&key=620c6b5c845e2a1c58db2232&metricsOrdinal=1&metricsResultType=substance&ordinal=1&resultType=substance&resultView=DETAIL&sortBy=relevance&sortOrder=descending&state=searchDetail.substance&uiContext=365&uiSubContext=551&uriForDetails=substance%2Fpt%2F29106498) |  |
| 20 | Vitexin 2''-O-rhamnoside | 64820-99-1 |  |
| 21 | Tetrandrine | [518-34-3](https://scifinder-n.cas.org/navigate/?appId=ee41cf3e-f4aa-402e-a3f1-da33fb255628&backKey=620c6b89845e2a1c58db2291&backToPage=1&contentUri=substance%2Fpt%2F518343&key=620c6b89845e2a1c58db2291&metricsOrdinal=1&metricsResultType=substance&ordinal=1&resultType=substance&resultView=DETAIL&sortBy=relevance&sortOrder=descending&state=searchDetail.substance&uiContext=365&uiSubContext=551&uriForDetails=substance%2Fpt%2F518343) |  |
| 22 | Licochalcone A | [58749-22-7](https://scifinder-n.cas.org/navigate/?appId=ee41cf3e-f4aa-402e-a3f1-da33fb255628&backKey=620c6caf845e2a1c58db2508&backToPage=1&contentUri=substance%2Fpt%2F58749227&key=620c6caf845e2a1c58db2508&metricsOrdinal=1&metricsResultType=substance&ordinal=1&resultType=substance&resultView=DETAIL&sortBy=relevance&sortOrder=descending&state=searchDetail.substance&uiContext=365&uiSubContext=551&uriForDetails=substance%2Fpt%2F58749227) |  |
| 23 | Puerarin | [3681-99-0](https://scifinder-n.cas.org/navigate/?appId=ee41cf3e-f4aa-402e-a3f1-da33fb255628&backKey=620c6cdc845e2a1c58db256a&backToPage=1&contentUri=substance%2Fpt%2F3681990&key=620c6cdc845e2a1c58db256a&metricsOrdinal=1&metricsResultType=substance&ordinal=1&resultType=substance&resultView=DETAIL&sortBy=relevance&sortOrder=descending&state=searchDetail.substance&uiContext=365&uiSubContext=551&uriForDetails=substance%2Fpt%2F3681990) |  |
| 24 | Hyperoside | [482-36-0](https://scifinder-n.cas.org/navigate/?appId=ee41cf3e-f4aa-402e-a3f1-da33fb255628&backKey=620c6cf6845e2a1c58db259f&backToPage=1&contentUri=substance%2Fpt%2F482360&key=620c6cf6845e2a1c58db259f&metricsOrdinal=1&metricsResultType=substance&ordinal=1&resultType=substance&resultView=DETAIL&sortBy=relevance&sortOrder=descending&state=searchDetail.substance&uiContext=365&uiSubContext=551&uriForDetails=substance%2Fpt%2F482360) |  |
| 25 | Cucurbitacin B | [6199-67-3](https://scifinder-n.cas.org/navigate/?appId=ee41cf3e-f4aa-402e-a3f1-da33fb255628&backKey=620c6d0a845e2a1c58db25d0&backToPage=1&contentUri=substance%2Fpt%2F6199673&key=620c6d0a845e2a1c58db25d0&metricsOrdinal=1&metricsResultType=substance&ordinal=1&resultType=substance&resultView=DETAIL&sortBy=relevance&sortOrder=descending&state=searchDetail.substance&uiContext=365&uiSubContext=551&uriForDetails=substance%2Fpt%2F6199673) |  |
| 26 | 9''-Methyl salvianolate B | 1167424-31-8 |  |
| 27 | Neo-przewaquinone A | [630057-39-5](https://scifinder-n.cas.org/navigate/?appId=ee41cf3e-f4aa-402e-a3f1-da33fb255628&backKey=620c6d2b845e2a1c58db261d&backToPage=1&contentUri=substance%2Fpt%2F630057395&key=620c6d2b845e2a1c58db261d&metricsOrdinal=1&metricsResultType=substance&ordinal=1&resultType=substance&resultView=DETAIL&sortBy=relevance&sortOrder=descending&state=searchDetail.substance&uiContext=365&uiSubContext=551&uriForDetails=substance%2Fpt%2F630057395) |  |
| 28 | Salvianolic acid F | [158732-59-3](https://scifinder-n.cas.org/navigate/?appId=ee41cf3e-f4aa-402e-a3f1-da33fb255628&backKey=620c6c9b845e2a1c58db24db&backToPage=1&contentUri=substance%2Fpt%2F158732593&key=620c6c9b845e2a1c58db24db&metricsOrdinal=1&metricsResultType=substance&ordinal=1&resultType=substance&resultView=DETAIL&sortBy=relevance&sortOrder=descending&state=searchDetail.substance&uiContext=365&uiSubContext=551&uriForDetails=substance%2Fpt%2F158732593) |  |
| 29 | 9'''-Methyl salvianolate B | 1167424-32-9 |  |
| 30 | Chrysophanol-1-O-β-D-glucoside | 4839-60-5 |  |
| 31 | Chrysophanol-8-O-β-D-glucoside | 13241-28-6 |  |
| 32 | Chelidonine | 476-32-4 |  |
| 33 | Eupalinolide O | [2170228-67-6](https://scifinder-n.cas.org/navigate/?appId=ee41cf3e-f4aa-402e-a3f1-da33fb255628&backKey=620c6c3a845e2a1c58db2410&backToPage=1&contentUri=substance%2Fpt%2F2170228676&key=620c6c3a845e2a1c58db2410&metricsOrdinal=1&metricsResultType=substance&ordinal=1&resultType=substance&resultView=DETAIL&sortBy=relevance&sortOrder=descending&state=searchDetail.substance&uiContext=365&uiSubContext=551&uriForDetails=substance%2Fpt%2F2170228676) | 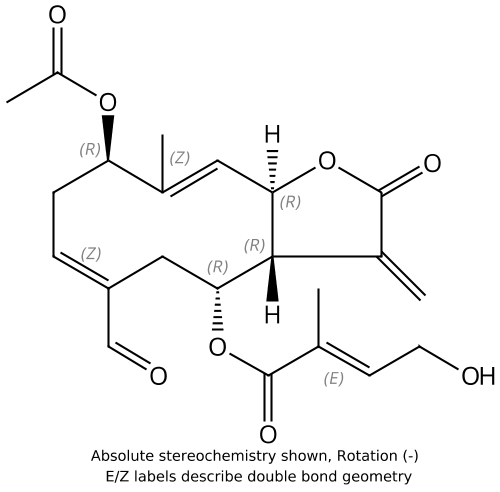 |
| 34 | Platycodin D | [58479-68-8](https://scifinder-n.cas.org/navigate/?appId=ee41cf3e-f4aa-402e-a3f1-da33fb255628&backKey=620c6c22845e2a1c58db23e2&backToPage=1&contentUri=substance%2Fpt%2F58479688&key=620c6c22845e2a1c58db23e2&metricsOrdinal=1&metricsResultType=substance&ordinal=1&resultType=substance&resultView=DETAIL&sortBy=relevance&sortOrder=descending&state=searchDetail.substance&uiContext=365&uiSubContext=551&uriForDetails=substance%2Fpt%2F58479688) |  |
| 35 | Ginsenoside F3 | [62025-50-7](https://scifinder-n.cas.org/navigate/?appId=ee41cf3e-f4aa-402e-a3f1-da33fb255628&backKey=620c6bee845e2a1c58db2366&backToPage=1&contentUri=substance%2Fpt%2F62025507&key=620c6bee845e2a1c58db2366&metricsOrdinal=1&metricsResultType=substance&ordinal=1&resultType=substance&resultView=DETAIL&sortBy=relevance&sortOrder=descending&state=searchDetail.substance&uiContext=365&uiSubContext=551&uriForDetails=substance%2Fpt%2F62025507) |  |
| 36 | Isolinderalactone | [957-66-4](https://scifinder-n.cas.org/navigate/?appId=ee41cf3e-f4aa-402e-a3f1-da33fb255628&backKey=620c6be0845e2a1c58db2344&backToPage=1&contentUri=substance%2Fpt%2F957664&key=620c6be0845e2a1c58db2344&metricsOrdinal=1&metricsResultType=substance&ordinal=1&resultType=substance&resultView=DETAIL&sortBy=relevance&sortOrder=descending&state=searchDetail.substance&uiContext=365&uiSubContext=551&uriForDetails=substance%2Fpt%2F957664) |  |
| 37 | Alantolactone | [546-43-0](https://scifinder-n.cas.org/navigate/?appId=ee41cf3e-f4aa-402e-a3f1-da33fb255628&backKey=620c6bb1845e2a1c58db22dd&backToPage=1&contentUri=substance%2Fpt%2F546430&key=620c6bb1845e2a1c58db22dd&metricsOrdinal=1&metricsResultType=substance&ordinal=1&resultType=substance&resultView=DETAIL&sortBy=relevance&sortOrder=descending&state=searchDetail.substance&uiContext=365&uiSubContext=551&uriForDetails=substance%2Fpt%2F546430) |  |
| 38 | Paris Saponin II | [50773-42-7](https://scifinder-n.cas.org/navigate/?appId=ee41cf3e-f4aa-402e-a3f1-da33fb255628&backKey=620c6bca845e2a1c58db2311&backToPage=1&contentUri=substance%2Fpt%2F50773427&key=620c6bca845e2a1c58db2311&metricsOrdinal=1&metricsResultType=substance&ordinal=1&resultType=substance&resultView=DETAIL&sortBy=relevance&sortOrder=descending&state=searchDetail.substance&uiContext=365&uiSubContext=551&uriForDetails=substance%2Fpt%2F50773427) |  |
| 39 | Eupalinolide A | [877822-40-7](https://scifinder-n.cas.org/navigate/?appId=ee41cf3e-f4aa-402e-a3f1-da33fb255628&backKey=620c6a3d845e2a1c58db1ffe&backToPage=1&contentUri=substance%2Fpt%2F877822407&key=620c6a3d845e2a1c58db1ffe&metricsOrdinal=1&metricsResultType=substance&ordinal=1&resultType=substance&resultView=DETAIL&sortBy=relevance&sortOrder=descending&state=searchDetail.substance&uiContext=365&uiSubContext=551&uriForDetails=substance%2Fpt%2F877822407) |  |
| 40 | Polyphyllin VI | [55916-51-3](https://scifinder-n.cas.org/navigate/?appId=ee41cf3e-f4aa-402e-a3f1-da33fb255628&backKey=620c6a4c845e2a1c58db2019&backToPage=1&contentUri=substance%2Fpt%2F55916513&key=620c6a4c845e2a1c58db2019&metricsOrdinal=1&metricsResultType=substance&ordinal=1&resultType=substance&resultView=DETAIL&sortBy=relevance&sortOrder=descending&state=searchDetail.substance&uiContext=365&uiSubContext=551&uriForDetails=substance%2Fpt%2F55916513) |  |
| 41 | Hispidulin | [1447-88-7](https://scifinder-n.cas.org/navigate/?appId=ee41cf3e-f4aa-402e-a3f1-da33fb255628&backKey=620c6a60845e2a1c58db2041&backToPage=1&contentUri=substance%2Fpt%2F1447887&key=620c6a60845e2a1c58db2041&metricsOrdinal=1&metricsResultType=substance&ordinal=1&resultType=substance&resultView=DETAIL&sortBy=relevance&sortOrder=descending&state=searchDetail.substance&uiContext=365&uiSubContext=551&uriForDetails=substance%2Fpt%2F1447887) |  |
| 42 | Chelerythrine | [34316-15-9](https://scifinder-n.cas.org/navigate/?appId=ee41cf3e-f4aa-402e-a3f1-da33fb255628&backKey=620c6a73845e2a1c58db2066&backToPage=1&contentUri=substance%2Fpt%2F34316159&key=620c6a73845e2a1c58db2066&metricsOrdinal=1&metricsResultType=substance&ordinal=1&resultType=substance&resultView=DETAIL&sortBy=relevance&sortOrder=descending&state=searchDetail.substance&uiContext=365&uiSubContext=551&uriForDetails=substance%2Fpt%2F34316159) |  |
| 43 | 20(S)-Ginsenoside Rh2 | [78214-33-2](https://scifinder-n.cas.org/navigate/?appId=ee41cf3e-f4aa-402e-a3f1-da33fb255628&backKey=620c6deb845e2a1c58db2793&backToPage=1&contentUri=substance%2Fpt%2F78214332&key=620c6deb845e2a1c58db2793&metricsOrdinal=1&metricsResultType=substance&ordinal=1&resultType=substance&resultView=DETAIL&sortBy=relevance&sortOrder=descending&state=searchDetail.substance&uiContext=365&uiSubContext=551&uriForDetails=substance%2Fpt%2F78214332) |  |
| 44 | (20R)-Ginsenoside Rh1 | [80952-71-2](https://scifinder-n.cas.org/navigate/?appId=ee41cf3e-f4aa-402e-a3f1-da33fb255628&backKey=620c6dff845e2a1c58db27b4&backToPage=1&contentUri=substance%2Fpt%2F80952712&key=620c6dff845e2a1c58db27b4&metricsOrdinal=1&metricsResultType=substance&ordinal=1&resultType=substance&resultView=DETAIL&sortBy=relevance&sortOrder=descending&state=searchDetail.substance&uiContext=365&uiSubContext=551&uriForDetails=substance%2Fpt%2F80952712) |  |
| 45 | Rhapontigenin | [500-65-2](https://scifinder-n.cas.org/navigate/?appId=ee41cf3e-f4aa-402e-a3f1-da33fb255628&backKey=620c6e0e845e2a1c58db27d3&backToPage=1&contentUri=substance%2Fpt%2F500652&key=620c6e0e845e2a1c58db27d3&metricsOrdinal=1&metricsResultType=substance&ordinal=1&resultType=substance&resultView=DETAIL&sortBy=relevance&sortOrder=descending&state=searchDetail.substance&uiContext=365&uiSubContext=551&uriForDetails=substance%2Fpt%2F500652) |  |
| 46 | Curcumol | [4871-97-0](https://scifinder-n.cas.org/navigate/?appId=ee41cf3e-f4aa-402e-a3f1-da33fb255628&backKey=620c6e1f845e2a1c58db27f2&backToPage=1&contentUri=substance%2Fpt%2F4871970&key=620c6e1f845e2a1c58db27f2&metricsOrdinal=1&metricsResultType=substance&ordinal=1&resultType=substance&resultView=DETAIL&sortBy=relevance&sortOrder=descending&state=searchDetail.substance&uiContext=365&uiSubContext=551&uriForDetails=substance%2Fpt%2F4871970) |  |
| 47 | Salvianolic acid B | [121521-90-2](https://scifinder-n.cas.org/navigate/?appId=ee41cf3e-f4aa-402e-a3f1-da33fb255628&backKey=620c6e30845e2a1c58db2814&backToPage=1&contentUri=substance%2Fpt%2F121521902&key=620c6e30845e2a1c58db2814&metricsOrdinal=1&metricsResultType=substance&ordinal=1&resultType=substance&resultView=DETAIL&sortBy=relevance&sortOrder=descending&state=searchDetail.substance&uiContext=365&uiSubContext=551&uriForDetails=substance%2Fpt%2F121521902) |  |
| 48 | Saponin C | 130551-41-6 | 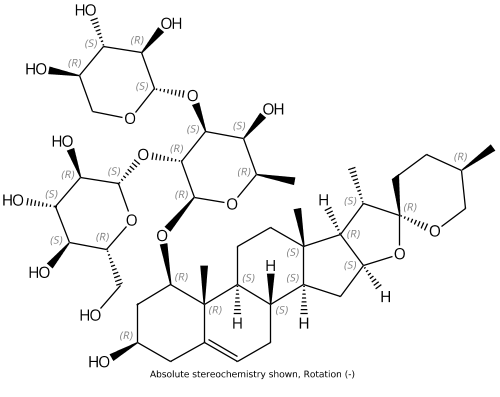 |
| 49 | Dimethyl lithospermate B | [875313-64-7](https://scifinder-n.cas.org/navigate/?appId=ee41cf3e-f4aa-402e-a3f1-da33fb255628&backKey=620c6e86845e2a1c58db28b7&backToPage=1&contentUri=substance%2Fpt%2F875313647&key=620c6e86845e2a1c58db28b7&metricsOrdinal=1&metricsResultType=substance&ordinal=1&resultType=substance&resultView=DETAIL&sortBy=relevance&sortOrder=descending&state=searchDetail.substance&uiContext=365&uiSubContext=551&uriForDetails=substance%2Fpt%2F875313647) |  |
| 50 | Salvianolic acid A | [96574-01-5](https://scifinder-n.cas.org/navigate/?appId=ee41cf3e-f4aa-402e-a3f1-da33fb255628&backKey=620c6e96845e2a1c58db28d2&backToPage=1&contentUri=substance%2Fpt%2F96574015&key=620c6e96845e2a1c58db28d2&metricsOrdinal=1&metricsResultType=substance&ordinal=1&resultType=substance&resultView=DETAIL&sortBy=relevance&sortOrder=descending&state=searchDetail.substance&uiContext=365&uiSubContext=551&uriForDetails=substance%2Fpt%2F96574015) |  |
| 51 | Engeletin | [572-31-6](https://scifinder-n.cas.org/navigate/?appId=ee41cf3e-f4aa-402e-a3f1-da33fb255628&backKey=620c6ebb845e2a1c58db2910&backToPage=1&contentUri=substance%2Fpt%2F572316&key=620c6ebb845e2a1c58db2910&metricsOrdinal=1&metricsResultType=substance&ordinal=1&resultType=substance&resultView=DETAIL&sortBy=relevance&sortOrder=descending&state=searchDetail.substance&uiContext=365&uiSubContext=551&uriForDetails=substance%2Fpt%2F572316) |  |
| 52 | Gomisin G | [62956-48-3](https://scifinder-n.cas.org/navigate/?appId=5ec88e69-8f43-4d64-a00f-ad33dd7ca2bf&backKey=620c7953845e2a1c58db3b97&backToPage=1&contentUri=substance%2Fpt%2F62956483&key=620c7953845e2a1c58db3b97&metricsOrdinal=1&metricsResultType=substance&ordinal=1&resultType=substance&resultView=DETAIL&sortBy=relevance&sortOrder=descending&state=searchDetail.substance&uiContext=365&uiSubContext=551&uriForDetails=substance%2Fpt%2F62956483) |  |
| 53 | Gomisin J | [66280-25-9](https://scifinder-n.cas.org/navigate/?appId=5ec88e69-8f43-4d64-a00f-ad33dd7ca2bf&backKey=620c7968845e2a1c58db3bc0&backToPage=1&contentUri=substance%2Fpt%2F66280259&key=620c7968845e2a1c58db3bc0&metricsOrdinal=1&metricsResultType=substance&ordinal=1&resultType=substance&resultView=DETAIL&sortBy=relevance&sortOrder=descending&state=searchDetail.substance&uiContext=365&uiSubContext=551&uriForDetails=substance%2Fpt%2F66280259) |  |
| 54 | Platycoside G1 | [849758-42-5](https://scifinder-n.cas.org/navigate/?appId=5ec88e69-8f43-4d64-a00f-ad33dd7ca2bf&backKey=620c7979845e2a1c58db3bdf&backToPage=1&contentUri=substance%2Fpt%2F849758425&key=620c7979845e2a1c58db3bdf&metricsOrdinal=1&metricsResultType=substance&ordinal=1&resultType=substance&resultView=DETAIL&sortBy=relevance&sortOrder=descending&state=searchDetail.substance&uiContext=365&uiSubContext=551&uriForDetails=substance%2Fpt%2F849758425) |  |
| 55 | Gypenoside LI | [94987-10-7](https://scifinder-n.cas.org/navigate/?appId=5ec88e69-8f43-4d64-a00f-ad33dd7ca2bf&backKey=620c798b845e2a1c58db3bff&backToPage=1&contentUri=substance%2Fpt%2F94987107&key=620c798b845e2a1c58db3bff&metricsOrdinal=1&metricsResultType=substance&ordinal=1&resultType=substance&resultView=DETAIL&sortBy=relevance&sortOrder=descending&state=searchDetail.substance&uiContext=365&uiSubContext=551&uriForDetails=substance%2Fpt%2F94987107) | 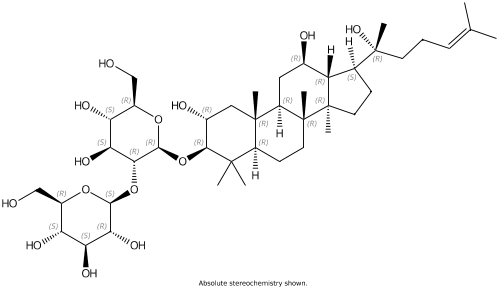 |
| 56 | Bacopaside IV | [155545-03-2](https://scifinder-n.cas.org/navigate/?appId=5ec88e69-8f43-4d64-a00f-ad33dd7ca2bf&backKey=620c79a0845e2a1c58db3c23&backToPage=1&contentUri=substance%2Fpt%2F155545032&key=620c79a0845e2a1c58db3c23&metricsOrdinal=1&metricsResultType=substance&ordinal=1&resultType=substance&resultView=DETAIL&sortBy=relevance&sortOrder=descending&state=searchDetail.substance&uiContext=365&uiSubContext=551&uriForDetails=substance%2Fpt%2F155545032) | 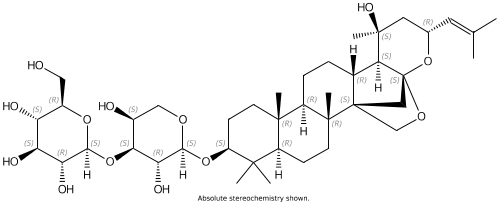 |
| 57 | Bacopasaponin C | [178064-13-6](https://scifinder-n.cas.org/navigate/?appId=5ec88e69-8f43-4d64-a00f-ad33dd7ca2bf&backKey=620c79b0845e2a1c58db3c42&backToPage=1&contentUri=substance%2Fpt%2F178064136&key=620c79b0845e2a1c58db3c42&metricsOrdinal=1&metricsResultType=substance&ordinal=1&resultType=substance&resultView=DETAIL&sortBy=relevance&sortOrder=descending&state=searchDetail.substance&uiContext=365&uiSubContext=551&uriForDetails=substance%2Fpt%2F178064136) |  |
| 58 | Bacopaside VII | [94443-88-6](https://scifinder-n.cas.org/navigate/?appId=5ec88e69-8f43-4d64-a00f-ad33dd7ca2bf&backKey=620c79c4845e2a1c58db3c6d&backToPage=1&contentUri=substance%2Fpt%2F94443886&key=620c79c4845e2a1c58db3c6d&metricsOrdinal=1&metricsResultType=substance&ordinal=1&resultType=substance&resultView=DETAIL&sortBy=relevance&sortOrder=descending&state=searchDetail.substance&uiContext=365&uiSubContext=551&uriForDetails=substance%2Fpt%2F94443886) |  |
| 59 | Songorine | 509-24-0 |  |
| 60 | 6-Hydroxykaempferol 3-O-*β*-D-glucoside | 145134-61-8 | 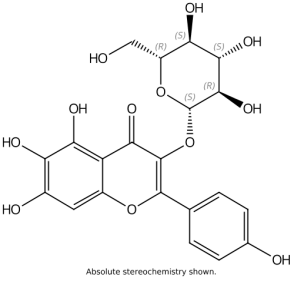 |
| 61 | Lycopene | [502-65-8](https://scifinder-n.cas.org/navigate/?appId=5ec88e69-8f43-4d64-a00f-ad33dd7ca2bf&backKey=620c7a6b845e2a1c58db3dd0&backToPage=1&contentUri=substance%2Fpt%2F502658&key=620c7a6b845e2a1c58db3dd0&metricsOrdinal=1&metricsResultType=substance&ordinal=1&resultType=substance&resultView=DETAIL&sortBy=relevance&sortOrder=descending&state=searchDetail.substance&uiContext=365&uiSubContext=551&uriForDetails=substance%2Fpt%2F502658) | 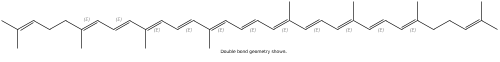 |
| 62 | Sodium Houttuyfonate | 1847-58-1 |  |
| 63 | 6-Hydroxykaempferol 3-*β*-rutinoside | [205527-00-0](https://scifinder-n.cas.org/navigate/?appId=5ec88e69-8f43-4d64-a00f-ad33dd7ca2bf&backKey=620c7a93845e2a1c58db3e1d&backToPage=1&contentUri=substance%2Fpt%2F205527000&key=620c7a93845e2a1c58db3e1d&metricsOrdinal=1&metricsResultType=substance&ordinal=1&resultType=substance&resultView=DETAIL&sortBy=relevance&sortOrder=descending&state=searchDetail.substance&uiContext=365&uiSubContext=551&uriForDetails=substance%2Fpt%2F205527000) | 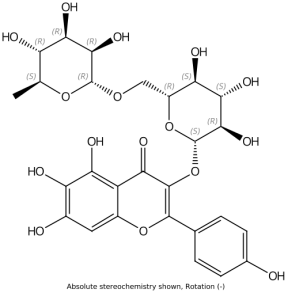 |
| 64 | Hydroxysafflor yellow A | [78281-02-4](https://scifinder-n.cas.org/navigate/?appId=ee41cf3e-f4aa-402e-a3f1-da33fb255628&backKey=620c6e4a845e2a1c58db2845&backToPage=1&contentUri=substance%2Fpt%2F78281024&key=620c6e4a845e2a1c58db2845&metricsOrdinal=1&metricsResultType=substance&ordinal=1&resultType=substance&resultView=DETAIL&sortBy=relevance&sortOrder=descending&state=searchDetail.substance&uiContext=365&uiSubContext=551&uriForDetails=substance%2Fpt%2F78281024) |  |
| 65 | Tanshinone I | [568-73-0](https://scifinder-n.cas.org/navigate/?appId=ee41cf3e-f4aa-402e-a3f1-da33fb255628&backKey=620c6dd6845e2a1c58db276b&backToPage=1&contentUri=substance%2Fpt%2F568730&key=620c6dd6845e2a1c58db276b&metricsOrdinal=1&metricsResultType=substance&ordinal=1&resultType=substance&resultView=DETAIL&sortBy=relevance&sortOrder=descending&state=searchDetail.substance&uiContext=365&uiSubContext=551&uriForDetails=substance%2Fpt%2F568730) |  |
| 66 | Deoxyandrographolide | 4176-97-0 |  |
| 67 | Oleanolic acid | 508-02-1 |  |
| 68 | Taxifolin | 480-18-2 |  |
| 69 | Salvianolic acid C | 115841-09-3 |  |
| 70 | Fumaric acid | [110-17-8](https://scifinder-n.cas.org/navigate/?appId=ee41cf3e-f4aa-402e-a3f1-da33fb255628&backKey=620c6d7f845e2a1c58db26c3&backToPage=1&contentUri=substance%2Fpt%2F110178&key=620c6d7f845e2a1c58db26c3&metricsOrdinal=1&metricsResultType=substance&ordinal=1&resultType=substance&resultView=DETAIL&sortBy=relevance&sortOrder=descending&state=searchDetail.substance&uiContext=365&uiSubContext=551&uriForDetails=substance%2Fpt%2F110178) | 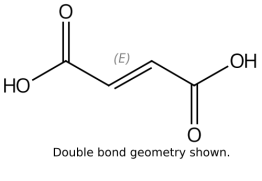 |
| 71 | Deoxyschizandrin | 61281-38-7 | 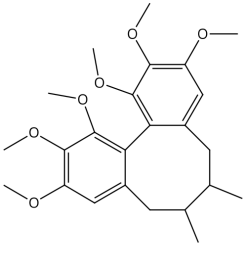 |
| 72 | Physcion 8-*β*-D-glucoside | 23451-01-6 |  |
| 73 | Genistin | [529-59-9](https://scifinder-n.cas.org/navigate/?appId=ee41cf3e-f4aa-402e-a3f1-da33fb255628&backKey=620c6d4c845e2a1c58db2662&backToPage=1&contentUri=substance%2Fpt%2F529599&key=620c6d4c845e2a1c58db2662&metricsOrdinal=1&metricsResultType=substance&ordinal=1&resultType=substance&resultView=DETAIL&sortBy=relevance&sortOrder=descending&state=searchDetail.substance&uiContext=365&uiSubContext=551&uriForDetails=substance%2Fpt%2F529599) |  |
| 74 | Notoginsenoside R1 | [80418-24-2](https://scifinder-n.cas.org/navigate/?appId=ee41cf3e-f4aa-402e-a3f1-da33fb255628&backKey=620c6b1d845e2a1c58db21c2&backToPage=1&contentUri=substance%2Fpt%2F80418242&key=620c6b1d845e2a1c58db21c2&metricsOrdinal=1&metricsResultType=substance&ordinal=1&resultType=substance&resultView=DETAIL&sortBy=relevance&sortOrder=descending&state=searchDetail.substance&uiContext=365&uiSubContext=551&uriForDetails=substance%2Fpt%2F80418242) |  |
| 75 | Dihydrotanshinone I | [87205-99-0](https://scifinder-n.cas.org/navigate/?appId=ee41cf3e-f4aa-402e-a3f1-da33fb255628&backKey=620c6b07845e2a1c58db2194&backToPage=1&contentUri=substance%2Fpt%2F87205990&key=620c6b07845e2a1c58db2194&metricsOrdinal=1&metricsResultType=substance&ordinal=1&resultType=substance&resultView=DETAIL&sortBy=relevance&sortOrder=descending&state=searchDetail.substance&uiContext=365&uiSubContext=551&uriForDetails=substance%2Fpt%2F87205990) |  |
| 76 | Kaempferol-3-O-(2"-O-β-D- glucopyl)-β-D–rutinoside | 55696-58-7 |  |
| 77 | Kaempferol 3-gentiobioside | [22149-35-5](https://scifinder-n.cas.org/navigate/?appId=ee41cf3e-f4aa-402e-a3f1-da33fb255628&backKey=620c6ac1845e2a1c58db2108&backToPage=1&contentUri=substance%2Fpt%2F22149355&key=620c6ac1845e2a1c58db2108&metricsOrdinal=1&metricsResultType=substance&ordinal=1&resultType=substance&resultView=DETAIL&sortBy=relevance&sortOrder=descending&state=searchDetail.substance&uiContext=365&uiSubContext=551&uriForDetails=substance%2Fpt%2F22149355) |  |
| 78 | Schisandrol B | [58546-54-6](https://scifinder-n.cas.org/navigate/?appId=ee41cf3e-f4aa-402e-a3f1-da33fb255628&backKey=620c5de8845e2a1c58db05b6&backToPage=1&contentUri=substance%2Fpt%2F58546546&key=620c5de8845e2a1c58db05b6&metricsOrdinal=1&metricsResultType=substance&ordinal=1&resultType=substance&resultView=DETAIL&sortBy=relevance&sortOrder=descending&state=searchDetail.substance&uiContext=365&uiSubContext=551&uriForDetails=substance%2Fpt%2F58546546) |  |
| 79 | Ellagic acid | [476-66-4](https://scifinder-n.cas.org/navigate/?appId=ee41cf3e-f4aa-402e-a3f1-da33fb255628&backKey=620c6ae5845e2a1c58db214a&backToPage=1&contentUri=substance%2Fpt%2F476664&key=620c6ae5845e2a1c58db214a&metricsOrdinal=1&metricsResultType=substance&ordinal=1&resultType=substance&resultView=DETAIL&sortBy=relevance&sortOrder=descending&state=searchDetail.substance&uiContext=365&uiSubContext=551&uriForDetails=substance%2Fpt%2F476664) |  |
| 80 | Salvianic acid A | 76822-21-4 |  |
| 81 | Methyl caffeate acid | 3843-74-1 |  |
| 82 | Asecin B | 26339-90-2 |  |
| 83 | Higenamine | 5843-65-2 | 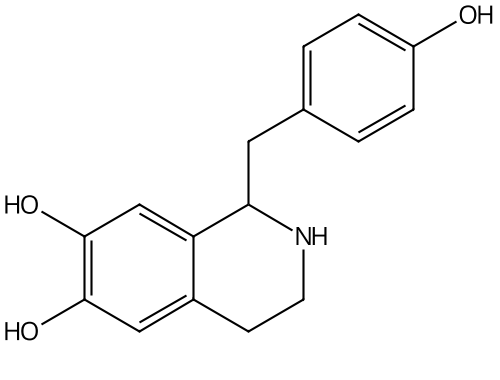 |
| 84 | Isomucronulatol | [64474-51-7](https://scifinder-n.cas.org/navigate/?appId=ee41cf3e-f4aa-402e-a3f1-da33fb255628&backKey=620c69be845e2a1c58db1eee&backToPage=1&contentUri=substance%2Fpt%2F64474517&key=620c69be845e2a1c58db1eee&metricsOrdinal=1&metricsResultType=substance&ordinal=1&resultType=substance&resultView=DETAIL&sortBy=relevance&sortOrder=descending&state=searchDetail.substance&uiContext=365&uiSubContext=551&uriForDetails=substance%2Fpt%2F64474517) |  |
| 85 | Dehydrocorydaline | [30045-16-0](https://scifinder-n.cas.org/navigate/?appId=ee41cf3e-f4aa-402e-a3f1-da33fb255628&backKey=620c69a8845e2a1c58db1ec1&backToPage=1&contentUri=substance%2Fpt%2F30045160&key=620c69a8845e2a1c58db1ec1&metricsOrdinal=1&metricsResultType=substance&ordinal=1&resultType=substance&resultView=DETAIL&sortBy=relevance&sortOrder=descending&state=searchDetail.substance&uiContext=365&uiSubContext=551&uriForDetails=substance%2Fpt%2F30045160) | 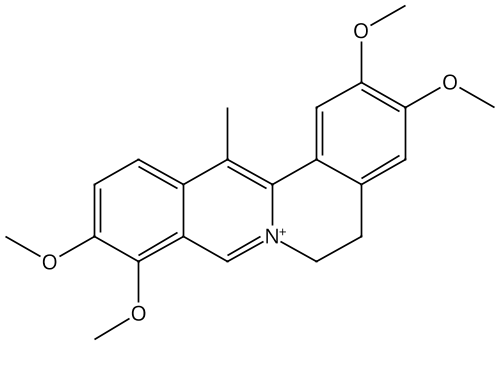 |
| 86 | 7-Epi-10-deacetyltaxol | 78454-17-8 | 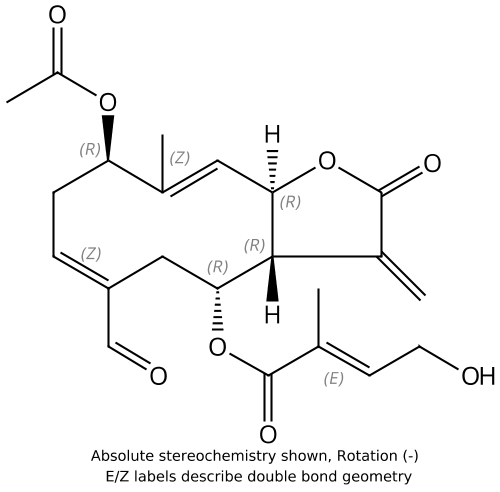 |
| 87 | Hydroxy Sprengerinin | 1111088-89-1 |  |
| 88 | 7-Epitaxol | [105454-04-4](https://scifinder-n.cas.org/navigate/?appId=ee41cf3e-f4aa-402e-a3f1-da33fb255628&backKey=620c5e2d845e2a1c58db0651&backToPage=1&contentUri=substance%2Fpt%2F105454044&key=620c5e2d845e2a1c58db0651&metricsOrdinal=1&metricsResultType=substance&ordinal=1&resultType=substance&resultView=DETAIL&sortBy=relevance&sortOrder=descending&state=searchDetail.substance&uiContext=365&uiSubContext=551&uriForDetails=substance%2Fpt%2F105454044) |  |
| 89 | Piceatannol | [10083-24-6](https://scifinder-n.cas.org/navigate/?appId=ee41cf3e-f4aa-402e-a3f1-da33fb255628&backKey=620c5d0c845e2a1c58db03e8&backToPage=1&contentUri=substance%2Fpt%2F10083246&key=620c5d0c845e2a1c58db03e8&metricsOrdinal=1&metricsResultType=substance&ordinal=1&resultType=substance&resultView=DETAIL&sortBy=relevance&sortOrder=descending&state=searchDetail.substance&uiContext=365&uiSubContext=551&uriForDetails=substance%2Fpt%2F10083246) |  |
| 90 | (-)-Epigallocatechin gallate | [989-51-5](https://scifinder-n.cas.org/navigate/?appId=ee41cf3e-f4aa-402e-a3f1-da33fb255628&backKey=620c5cea845e2a1c58db0396&backToPage=1&contentUri=substance%2Fpt%2F989515&key=620c5cea845e2a1c58db0396&metricsOrdinal=1&metricsResultType=substance&ordinal=1&resultType=substance&resultView=DETAIL&sortBy=relevance&sortOrder=descending&state=searchDetail.substance&uiContext=365&uiSubContext=551&uriForDetails=substance%2Fpt%2F989515) |  |
| 91 | 14-Deoxy-12-ethoxyl andrographolide | 141973-46-8 | 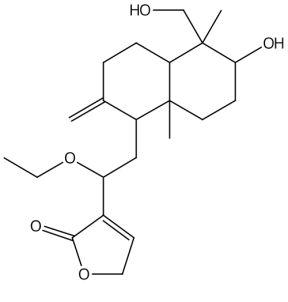 |
| 92 | Angelic acid | [565-63-9](https://scifinder-n.cas.org/navigate/?appId=ee41cf3e-f4aa-402e-a3f1-da33fb255628&backKey=620c5ca2845e2a1c58db02f0&backToPage=1&contentUri=substance%2Fpt%2F565639&key=620c5ca2845e2a1c58db02f0&metricsOrdinal=1&metricsResultType=substance&ordinal=1&resultType=substance&resultView=DETAIL&sortBy=relevance&sortOrder=descending&state=searchDetail.substance&uiContext=365&uiSubContext=551&uriForDetails=substance%2Fpt%2F565639) |  |
| 93 | Sodium Danshensu | [81075-52-7](https://scifinder-n.cas.org/navigate/?appId=ee41cf3e-f4aa-402e-a3f1-da33fb255628&backKey=620c5c8b845e2a1c58db02bd&backToPage=1&contentUri=substance%2Fpt%2F81075527&key=620c5c8b845e2a1c58db02bd&metricsOrdinal=1&metricsResultType=substance&ordinal=1&resultType=substance&resultView=DETAIL&sortBy=relevance&sortOrder=descending&state=searchDetail.substance&uiContext=365&uiSubContext=551&uriForDetails=substance%2Fpt%2F81075527) | 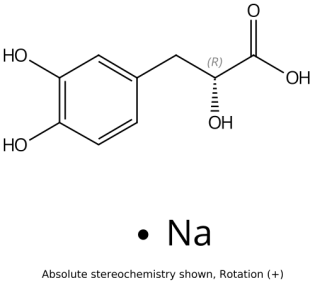 |
| 94 | Ginsenoside Rh1 | [63223-86-9](https://scifinder-n.cas.org/navigate/?appId=ee41cf3e-f4aa-402e-a3f1-da33fb255628&backKey=620c5c79845e2a1c58db028c&backToPage=1&contentUri=substance%2Fpt%2F63223869&key=620c5c79845e2a1c58db028c&metricsOrdinal=1&metricsResultType=substance&ordinal=1&resultType=substance&resultView=DETAIL&sortBy=relevance&sortOrder=descending&state=searchDetail.substance&uiContext=365&uiSubContext=551&uriForDetails=substance%2Fpt%2F63223869) |  |
| 95 | Ginsenoside Rk3 | [364779-15-7](https://scifinder-n.cas.org/navigate/?appId=ee41cf3e-f4aa-402e-a3f1-da33fb255628&backKey=620c5c66845e2a1c58db0269&backToPage=1&contentUri=substance%2Fpt%2F364779157&key=620c5c66845e2a1c58db0269&metricsOrdinal=1&metricsResultType=substance&ordinal=1&resultType=substance&resultView=DETAIL&sortBy=relevance&sortOrder=descending&state=searchDetail.substance&uiContext=365&uiSubContext=551&uriForDetails=substance%2Fpt%2F364779157) |  |
| 96 | Pterostilbene | [537-42-8](https://scifinder-n.cas.org/navigate/?appId=ee41cf3e-f4aa-402e-a3f1-da33fb255628&backKey=620c5c50845e2a1c58db0237&backToPage=1&contentUri=substance%2Fpt%2F537428&key=620c5c50845e2a1c58db0237&metricsOrdinal=1&metricsResultType=substance&ordinal=1&resultType=substance&resultView=DETAIL&sortBy=relevance&sortOrder=descending&state=searchDetail.substance&uiContext=365&uiSubContext=551&uriForDetails=substance%2Fpt%2F537428) |  |
| 97 | Lutein | [127-40-2](https://scifinder-n.cas.org/navigate/?appId=ee41cf3e-f4aa-402e-a3f1-da33fb255628&backKey=620c5c14845e2a1c58db01c0&backToPage=1&contentUri=substance%2Fpt%2F127402&key=620c5c14845e2a1c58db01c0&metricsOrdinal=1&metricsResultType=substance&ordinal=1&resultType=substance&resultView=DETAIL&sortBy=relevance&sortOrder=descending&state=searchDetail.substance&uiContext=365&uiSubContext=551&uriForDetails=substance%2Fpt%2F127402) | 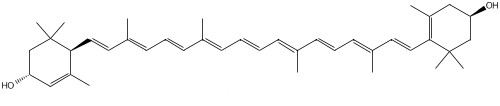 |
| 98 | Tanshinone IIA | 568-72-9 |  |
| 99 | 8-ethoxylyunnaconitine | 110011-77-3 |  |
| 100 | (1α,6β,8ξ,14α,16β,17R)-20-Ethyl-6,14,16-trimethoxy-4-(methoxymeth yl)aconitane-1,7,8-triol | 509-18-2 |  |
| 101 | Przewaquinone C | 96839-29-1 |  |
| 102 | Gypenoside XVII | 80321-69-3 |  |
| 103 | digoxin | 20830-75-5 |  |
| 104 | Platycoside D3 | 67884-03-1 |  |
| 105 | talatisamine | 20501-56-8 |  |
